# Ultrasound-guided renal artery administration of angiopoietin-1 RNA therapy slows the progression of preclinical WT1 glomerular disease

**DOI:** 10.1126/scitranslmed.adv1289

**Published:** 2026-08-12

**Authors:** Saif N Malik, Jennifer C Chandler, Ruhina Maeshima, Maria Kolatsi-Joannou, William J Mason, Lauren G Russell, Shunping Han, Yanis Harfouche, Daniyal J Jafree, Emily A E Moore, Hanna Gautier, Christina Katsiva, Reem Al-Saadi, Kathy Pritchard-Jones, Daniel J Stuckey, Andrew V Benest, David O Bates, Paul J Winyard, Aoife M Waters, Luigi Gnudi, Stephen L Hart, Tammy L Kalber, David A Long

**Affiliations:** 1 Developmental Biology and Cancer Research and Teaching Department, University College London Great Ormond Street Institute of Child Health; London, WC1N 1EH, UK; 2 UCL Centre for Kidney and Bladder Health, London, WC1N 1EH, UK; 3 Genetics and Genomic Medicine Research and Teaching Department, University College London Great Ormond Street Institute of Child Health; London, WC1N 1EH, UK; 4 Wellcome Sanger Institute, Wellcome Genome Campus, Hinxton, CB10 1RQ; 5 Centre for Advanced Biomedical Imaging, University College London; London, WC1E 6DD UK; 6 Department of Histopathology, Great Ormond Street Hospital for Children NHS Foundation Trust, London, WC1N 3JH, UK; 7 Endothelial Quiescence Group and Tumour and Vascular Biology Laboratories, Division of Cancer and Stem Cells, Centre for Cancer Sciences, School of Medicine, Biodiscovery Institute, University of Nottingham, Science Road, NG7 2RD, UK; 8 Pan-African Cancer Research Institute, University of Pretoria, Hatfield, 0028; 9 Unit for Metabolic Medicine, Section of Vascular Biology and Inflammation, School of Cardiovascular and Metabolic Medicine & Sciences, British Heart Foundation Centre for Excellence, Faculty of Life Sciences & Medicine, King’s College London, London, WC2R 2LS, UK

## Abstract

Mutations in the transcription factor *Wilms Tumour 1* (*WT1*) are one of the leading causes of congenital glomerular disease, characterised by severe urinary protein loss and glomerular scarring. Currently no disease modifying therapies exist for WT1 glomerulopathies and affected children rely on dialysis or kidney transplantation. We evaluated a new treatment in a mouse model with an orthologous human mutation in *Wt1* (*Wt1*^+*/R394W*^) that replicates the pathology of WT1 glomerulopathy. Lipid nanocomplexes were engineered to target integrin αvβ3 for efficient delivery of mRNA to primary podocytes and glomerular endothelial cells in vitro. A minimally invasive ultrasound-guided renal artery injection enabled precise and specific kidney localization in vivo, a finding not replicated by systemic administration. Nanocomplex-derived protein localized in glomeruli for up to seven days in healthy and diseased mice. This platform was then used to perform an interventional preclinical trial in *Wt1*^*+/R394W*^ mice, delivering angiopoietin-1 (*Angpt1*) mRNA, a vascular growth factor critical for glomerular health which is reduced in *Wt1*^*+/R394W*^ podocytes. *Angpt1* nanocomplex therapy reduced albuminuria, preserved glomerular endothelial integrity, prevented podocyte loss, and alleviated glomerulosclerosis in *Wt1*^*+/R394W*^ mice. These findings demonstrate the therapeutic potential of this targeted approach for WT1 glomerulopathy and provide a foundation for clinical translation to improve outcomes in children with glomerular disease.

## Introduction

Congenital glomerular diseases are among the most common diagnoses in childhood chronic kidney disease (CKD) (1). Over 80 monogenic causes of congenital glomerular disease have been identified, the majority of which implicate epithelial podocytes (2). Together with glomerular endothelial cells (gECs) and the glomerular basement membrane (GBM), podocytes form the glomerular permeability barrier, preventing molecules such as albumin from entering the urine (3). Variants in the podocyte transcription factor Wilms Tumour 1 (*WT1*) account for around 15% of congenital glomerular diseases, with patients commonly presenting with steroid-resistant nephrotic syndrome and diffuse mesangial sclerosis (DMS) (4). This includes a cohort with Denys-Drash syndrome (DDS, OMIM: 194080) that have a high risk of Wilms’ Tumour, with some patients presenting with disorders of sex development (5).

There are no disease-modifying therapies for WT1 glomerulopathies. This reflects not only the lack of treatment targets capable of slowing glomerular damage, but also the inadequacy of systemic administration routes that result in minimal kidney uptake (6, 7), necessitating higher therapeutic dosing and increasing the risk of adverse events. Additionally, the cellular complexity of the kidney means it is difficult to direct treatments to specific cells in the nephron (8). Glomerular therapies also need to overcome possible size and charge exclusion due to the presence of gEC fenestrations and podocyte slit diaphragms, as well as the negative charge of podocytes (9) and the GBM (10).

Glomerular structure and function are maintained by molecular signalling between podocytes and gECs (11). This communication is disrupted in adult glomerular diseases (11), as well as in *Wt1*^*+/R394W*^ mice (12), a genetically orthologous model of DDS which harbours the most commonly diagnosed *WT1* mutation (*WT1* c.1180C>T; p.R394W). *Wt1*^*+/R394W*^ mice replicate DDS glomerular pathology, with albuminuria and DMS but do not present with genital abnormalities nor Wilms’ Tumour (13). In the early stages of disease in *Wt1*^*+/R394W*^ mice, podocyte-gEC signalling is disrupted, prior to gEC loss, indicating that therapies targeting the vasculature may be beneficial (12). Angiopoietin-1 (ANGPT1) is a podocyte vascular growth factor (14), critical for glomerular health (15), through its maintenance of endothelial structure and permeability through tyrosine kinase with immunoglobulin-like and EGF-like domains 2 (TIE2) receptor activation (11, 16), and its support of perivascular-endothelial cell interactions (17). Reduced podocyte ANGPT1 expression occurs in preclinical models of adult glomerular disease (14, 18, 19). Therefore, considering the disruption in podocyte-gEC communication observed in *Wt1*^*+/R394W*^ mice, we hypothesised that ANGPT1 expression may be reduced in *Wt1*^*+/R394W*^ glomeruli, and that targeted *Angpt1* RNA therapy could slow disease progression in this context.

To test this, we designed a therapeutic approach to deliver *Angpt1* mRNA to glomeruli. Firstly, we predicted that local delivery would be more effective than systemic administration and evaluated this using a minimally invasive ultrasound-guided renal artery injection, with proven success in the administration of stem cells to mouse kidneys (20). Secondly, although studies incorporating glomerular cell-specific promoters (21) or targeting peptides (22) into adeno-associated viruses (AAV) have shown therapeutic promise, we adopted a non-viral approach, with the intention of reducing the risk of glomerular size or charge exclusion of our therapy. To do so, we encapsulated *Angpt1* mRNA into cationic lipid nanocomplexes that protect and stabilise negatively charged nucleic acids (23) and self-assemble into particles of different sizes (23). Finally, we reasoned that *Angpt1* therapy would be more effective if our nanocomplexes contained peptides which enhanced cellular uptake in podocytes and gECs. By incorporating these components, we sought to determine whether a glomerular targeted *Angpt1* nanocomplex, delivered locally through the renal artery, could slow progression of glomerular disease in *Wt1*^*+/R394W*^ mice.

## Results

### Nanocomplexes containing integrin αvβ3 targeting peptides improve podocyte and gEC transfection

Prior studies have shown nanocomplexes containing an integrin targeted cyclic Arg-Gly-Asp (RGD) peptide facilitate podocyte uptake (24). However, RGD binds multiple integrins (25) found in non-glomerular nephron cells (26), limiting the specificity of these nanocomplexes for treating glomerular disease. We reasoned that nanocomplexes containing targeting peptides binding to glomerular enriched integrins would be more effective. We identified integrin αvβ3 as a promising target reflecting its reported expression profile in podocytes and gECs (27–29).

**Fig. 1:**
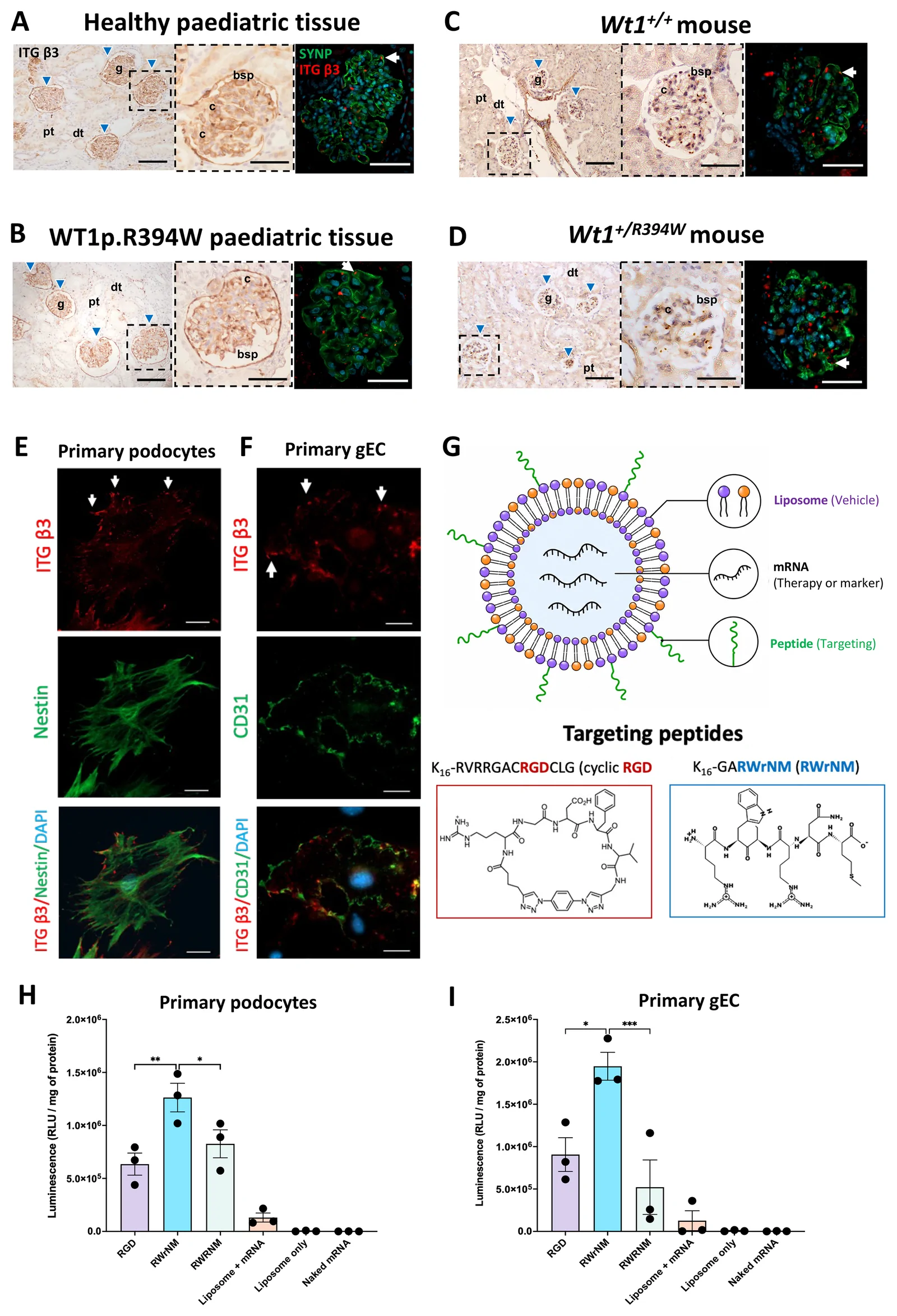
Nanocomplexes containing integrin αvβ3 targeting peptides enhance transfection of podocytes and gECs. (**A**) Kidney sections stained for integrin β3 (ITGβ3) by immunohistochemistry and immunofluorescence from healthy pediatric humans, (**B**) WT1p.R394W human biopsy, (**C**) *Wt1*^*+/+*^ mice, and (**D**) *Wt1*^*+/R394W*^ mice. Glomerulus (**g**), proximal tubule (**pt**), distal tubule (**dt**), capillary (**c**) and Bowman’s space (**bsp**). Blue arrowheads indicate ITGβ3 glomeruli. White arrows show ITGβ3 (red) adjacent to synaptopodin^+^ podocytes (green). Scale bars = 50 μm. **(E)** Primary murine podocytes labelled with nestin (green) and (**F**) gECs labelled with CD31 (green) show ITGβ3 (red) localization on plasma membrane, highlighted by white arrows. DAPI is blue. Scale bars = 5 μm. (**G**) Lipid nanocomplex formulated with mRNA, cationic liposome and targeting peptides. (**H**) Luminescence assays for primary murine podocytes and (**I**) gECs comparing uptake of luciferase mRNA nanocomplexes incorporating either RWrNM, RGD and RWRNM targeting peptides. For three independent experiments, cells were plated as *n* = 6 per group; mean values shown. Data represents mean ± SEM, (one-way ANOVA with Tukey’s multiple comparisons, *P≤0.05, **P≤0.01).

First, we determined the localization of integrin αvβ3 within healthy human kidneys. Kidney sections from two healthy pediatric donors were stained for integrin β3, selected to represent αvβ3 expression as integrin αv subunits form additional heterodimers in the kidney besides αvβ3, whereas β3 does not (29). β3 expression was enriched in healthy glomeruli compared with non-glomerular regions (Fig. 1A). This β3 glomerular enrichment was replicated (Fig. 1B) in biopsy tissue from a patient with the WT1p.R394W mutation, with DMS on histology (12). Glomerular enrichment of β3 was also observed in kidney sections of 8-week-old wild-type *Wt1*^*+/+*^ (Fig. 1C) and *Wt1*^*+/R394W*^ (Fig. 1D) mice, when glomerular scarring was present (12). Immunofluorescent co-staining for synaptopodin found β3 expression in close proximity to glomerular podocytes in healthy pediatric and WT1p.R394W patient biopsy tissue (Fig. 1A and B), a finding replicated in mouse kidneys (Fig. 1C and D). To examine this at the cellular level, β3 localization was assessed in wild-type primary WT1^+^/synaptopodin^+^ (fig. S1A to D) mouse podocytes (Fig. 1E) and cluster of differentiation 31 (CD31)^+^ gECs (Fig. 1F). In both cell types, β3 showed expression on the plasma membrane (Fig. 1E and F). β3 expression was also detected on the plasma membrane of diseased *Wt1*^*+/R394W*^ primary podocytes (fig. S2A) and significantly elevated mRNA expression was apparent in *Wt1*^*+/+*^ and *Wt1*^+*/R394W*^ primary podocytes compared with the whole kidney (fig. S2B; *P* = 0.003 and *P* = 0.008 respectively for *Wt1*^*+/+*^ and *Wt1*^*+/R394W*^ podocytes versus whole kidney), with no difference between genotype (*P* = 0.96). Together, these data demonstrated glomerular localization and enrichment of integrin β3 in podocytes and gECs, providing rationale to examine the efficacy of targeting αvβ3 for specific transfection of both cell types.

We manufactured lipid nanocomplexes based on formulations successfully used for delivery of nucleic acids to tumour and lung cells (23). We compared the transfection efficiency of nanocomplexes incorporating peptides (30) containing either cyclic RGD or RWrNM targeting motifs, on primary podocytes and gECs. The RWrNM motif, an RGD peptidomimetic, was chosen as it has higher affinity and specificity for αvβ3 compared to RGD when assessed in tumour cells (31). As an additional control, we included nanocomplexes with a RWRNM motif to evaluate the importance of the central arginine chirality (D- or L-Arginine) on integrin αvβ3 binding affinity. Each peptide was complexed with cationic liposomes, encapsulating luciferase mRNA (Fig. 1G). Initial experiments identified DOTMA:DOPE (C18) as the most efficient liposome in primary podocytes, and this was used for all subsequent experiments (fig. S3). RGD and RWrNM luciferase mRNA nanocomplex formulations had a respective mean size ± standard deviation of 202.1 nm ± 7.0 and 220 nm ± 1.0 (fig. S4A and B); polydispersity index (PDI) of 0.29 ± 0.01 and 0.25 ± 0.02 and zeta potential (ZP) of 38.1 ± 0.4 mV and 56.3 ± 0.5 mV (table S1).

RWrNM nanocomplexes elicited 2- and 1.5-fold significantly higher transfection rates in wild-type primary murine podocytes than RGD and RWRNM nanocomplexes respectively (Fig. 1H; RGD = 6.35 × 10^5^ ± 1.04 × 10^5^, RWrNM = 1.26 × 10^6^ ± 1.33 × 10^5^ and RWRNM = 8.27 × 10^5^ ± 1.28 × 10^5^ RLU/mg of protein, *P* = 0.004 and *P* = 0.04 for RWrNM versus RGD and RWRNM respectively). In primary murine gECs, RWrNM nanocomplexes elicited 2.1- and 3.7-fold significantly higher transfection rates than RGD and RWRNM respectively (Fig. 1I; RGD = 9.06 × 10^5^ ± 1.93 × 10^5^, RWrNM = 1.95 × 10^6^ ± 1.60 × 10^5^ and RWRNM = 5.21 × 10^5^ ± 4.26 × 10^5^ RLU/mg of protein, *P* = 0.01 and *P* < 0.001 for RWrNM versus RGD and RWRNM respectively). We also tested whether RWrNM nanocomplexes improved transfection in mutant *Wt1*^*+/R394W*^ podocytes isolated at 8 weeks of age during advanced disease (12) and found 6- and 3-fold significantly higher transfection efficiency than RGD and RWRNM nanocomplexes respectively (fig. S5A; RGD = 1.77 × 10^5^ ± 4.23 × 10^4^, RWrNM = 1.09 × 10^6^ ± 2.21 × 10^5^ and RWRNM = 3.23 × 10^5^ ± 1.71 × 10^5^ RLU/mg of protein, *P* = 0.0013 and *P* = 0.0054 for RWrNM versus RGD and RWRNM respectively). Finally, to determine whether the *Wt1* mutation and its associated phenotype affected nanocomplex uptake, we directly compared RWrNM nanocomplex uptake between wild-type and *Wt1*^*+/R394W*^ primary podocytes isolated at 8 weeks of age and found no significant difference (fig. S5B; *Wt1*^*+/+*^ = 2.69 × 10^6^ ± 9.40 × 10^5^ and *Wt1*^*+/R394W*^ = 3.03 × 10^6^ ± 1.06 × 10^6^, *P* = 0.81). Collectively, this demonstrates the efficacy of RWrNM for αvβ3-mediated transfection in primary podocytes and gECs in vitro.

### Ultrasound-guided renal artery injection facilitates kidney localization of RWrNM nanocomplexes in vivo

Next, we aimed to assess the in vivo biodistribution of RWrNM nanocomplexes containing 20 μg of luciferase mRNA (97.24 ± 2.19 % encapsulation efficiency) delivered through a minimally invasive, ultrasound-guided renal artery injection (20). Color doppler mode visualized kidney blood vessels allowing for direct injection into the renal artery without surgical intervention. The right kidney of 5-week-old female CD1 nude mice was injected and live whole-body, bioluminescent imaging (BLI) scans were taken 18 hours post injection, followed by ex vivo assessment of the injected kidney, non-injected control kidney, lungs and liver.

**Fig. 2:**
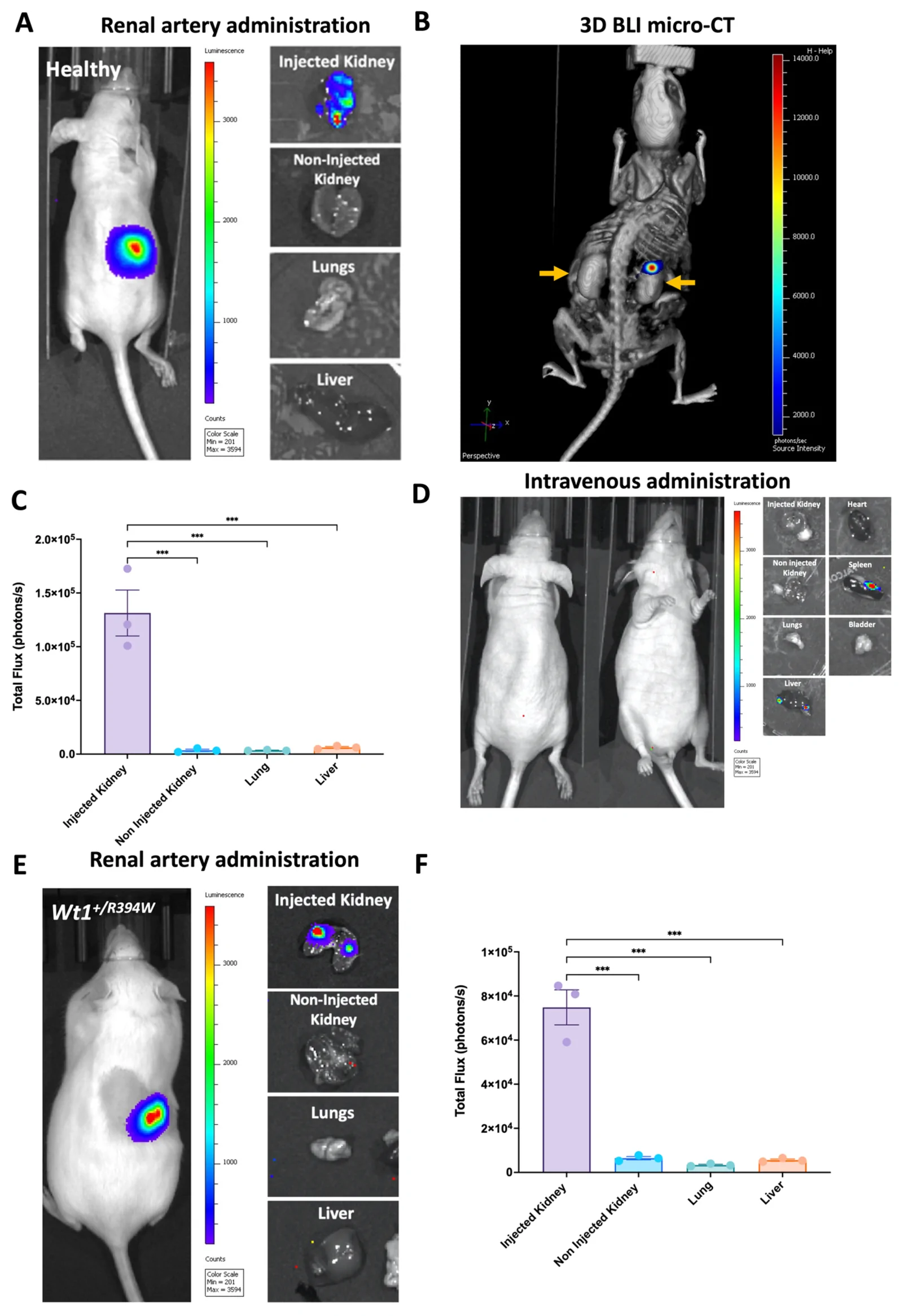
RWrNM peptide lipid nanocomplexes demonstrate specific kidney localization following ultrasound-guided renal artery injection. (**A**) CD1 mice (*n =* 3) injected with 20 μg luciferase mRNA RWrNM nanocomplexes into the renal artery of the right kidney under ultrasound guidance show specific kidney expression in BLI. (**B**) Kidney specificity (yellow arrows) of luciferase mRNA RWrNM nanocomplexes injected in the right kidney visualized after superimposing a diffuse luminescence imaging tomography 3D reconstruction and a micro-CT image. (**C**) Total flux for ex vivo organs isolated from CD1 mice injected with luciferase mRNA RWrNM nanocomplexes (*n =* 3). Each data point represents the total flux for one organ/mouse. Data represents mean ± SEM, (one-way ANOVA with Tukey’s multiple comparisons, ***P≤0.001). (**D**) Representative in vivo and ex vivo BLI images of two CD1 mice 18 hours post intravenous injection of 20 μg luciferase mRNA nanocomplexes. (**E**) Representative in vivo and ex vivo BLI of three *Wt1*^*+/R394W*^ mice post renal artery injection with 20 μg luciferase mRNA RWrNM nanocomplexes. (**F**) Quantification of total flux for each ex vivo organ from panel E. Colour scale in all BLI images represents intensity of total flux (photons/second) from highest (red) to lowest (purple) detected intensity. Data represents mean ± SEM, (one-way ANOVA with Tukey’s multiple comparisons, ***P≤0.001).

Whole body scans showed specific localization in the injected right kidney with no apparent signal from the rest of the body (Fig. 2A). Quantitative analysis of the total bioluminescence in each mouse revealed that 80.67 ± 6.48 % of the mean percentage signal was accounted for by the injected kidney (fig. S6). To validate live kidney localization, we combined three dimensional (3D) BLI with micro computed tomography (micro-CT) following renal artery injection of luciferase mRNA RWrNM nanocomplexes. The 3D BLI reconstruction superimposed onto a micro-CT scan of the mouse showed the BLI signal localized to the injected right kidney (Fig. 2B). Injected kidneys showed a significantly higher luminescent signal ex vivo (photons/second) than either the non-injected kidney (36.9-fold), lungs (37.6-fold) and liver (21.2-fold) (Fig. 2C; injected kidney = 1.31 × 10^5^ ± 2.15 × 10^4^, non-injected kidney = 3.55 × 10^3^ ± 1.01 × 10^3^, lung = 3.48 × 10^3^ ± 2.74 × 10^2^, liver = 6.18 × 10^3^ ± 8.67 × 10^2^, injected kidney versus all other organs *P* < 0.001).

To compare the biodistribution of locally and systemically delivered RWrNM nanocomplexes, we injected luciferase mRNA nanocomplexes at the same dose into the tail vein of female 5-week-old CD1 nude mice. Luminescent signal was not observed in any organ following whole body in vivo dorsal and ventral BLI scans. No signal was found in either kidney ex vivo (Fig. 2D) with bioluminescence only observed in the liver of one mouse (5.52 × 10^4^ photons per second) and spleen of both mice (8.68 × 10^4^ ± 6.13 × 10^3^ photons per second).

We also assessed biodistribution following renal artery injection in 8-week-old male *Wt1*^*+/R394W*^ mice. In all animals, the luminescent signal localized to the injected kidney in vivo and ex vivo (Fig. 2E). Although fur coverage restricted quantitative analysis of whole body bioluminescence, ex vivo measurements showed the injected kidney to have significantly higher luminescence than the non-injected kidney (12.1-fold), lung (23.3-fold) and liver (14.0-fold), as observed in the CD1 mice (Fig. 2F; injected kidney = 7.48 × 10^4^ ± 7.97 × 10^3^, non-injected kidney = 6.48 × 10^3^ ± 7.41 × 10^2^, lung = 3.36 × 10^3^ ± 3.35 × 10^2^, liver = 5.60 × 10^3^ ± 4.90 × 10^2^ photons/second, injected kidney versus all other organs *P* < 0.001). Together, this confirmed a suitable delivery technique to localize RWrNM nanocomplexes to the kidney in health and glomerular disease.

### Nanocomplex cargo is detected in kidney glomeruli up to 7 days post injection in *Wt1*^*+/+*^ and *Wt1*^*+/R394W*^ mice

Once kidney localization of the RWrNM nanocomplexes was evident, we aimed to determine glomerular uptake and the longevity of protein expression in vivo. To do so, we administered mCherry mRNA (20 μg) RWrNM nanocomplexes (mean size = 227.4 ± 5.9 nm (fig. S4C); mean PDI = 0.264 ± 0.015; mean ZP = 41.4 ± 0.7 mV; encapsulation efficiency = 97.68 ± 2.54 %) (table S1) into the right kidney of *Wt1*^+/+^ and *Wt1*^*+/R394W*^ mice at 4 and 8 weeks of age. These time points represent early and advanced disease in *Wt1*^*+/R394W*^ mice, with albuminuria but no overt histological damage at 4 weeks and elevated blood urea nitrogen (BUN) alongside glomerular scarring at 8 weeks (12).

**Fig. 3:**
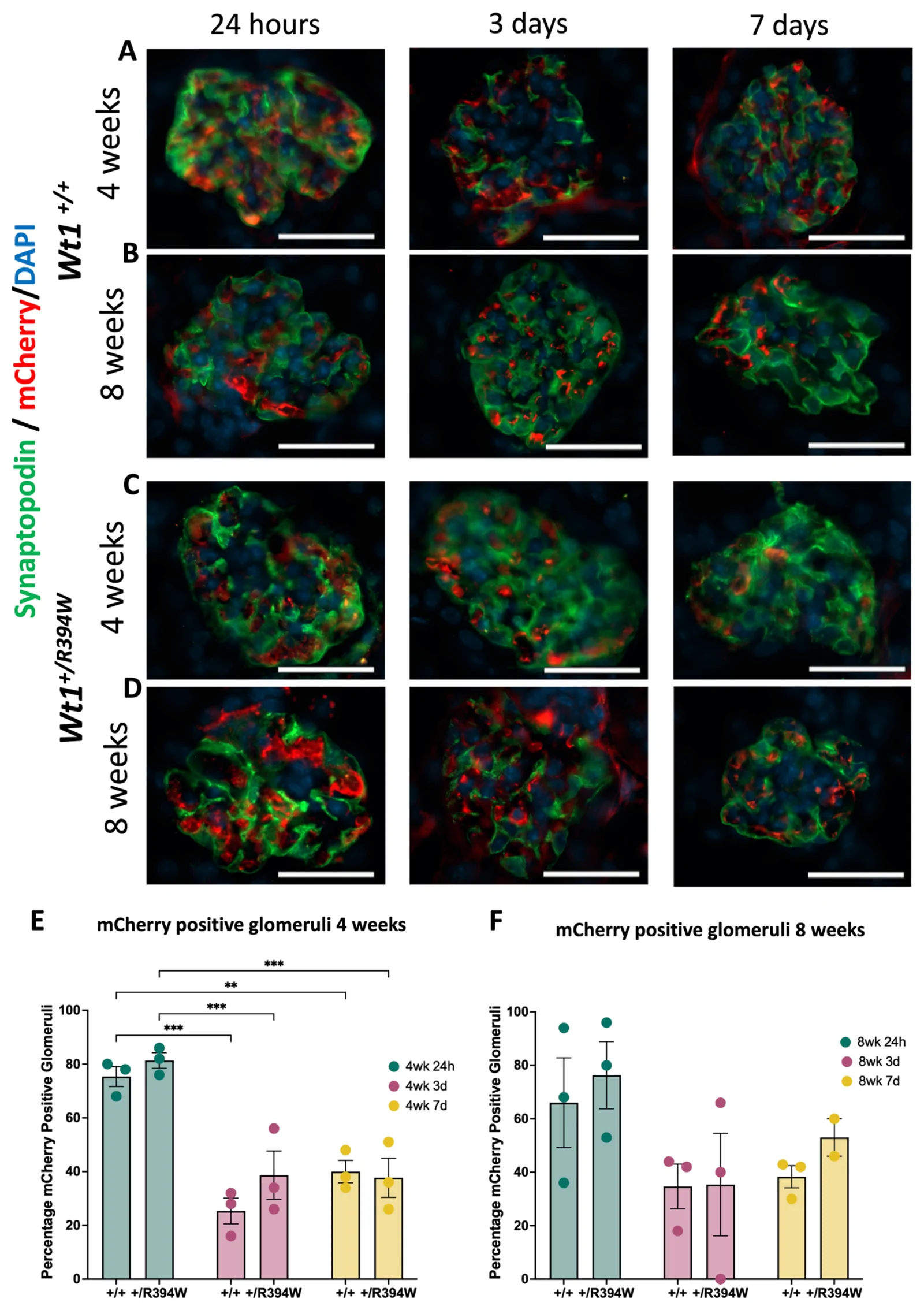
Nanocomplex mCherry cargo localizes to glomeruli and is expressed up to 7 days post injection in *Wt1*^*+/+*^ and *Wt1*^*+/R394W*^ mice. (**A**) Representative micrographs from three independent animals/group and time-point showing prominent expression of mCherry (red) in the injected right kidney of *Wt1*^*+/+*^ animals at 4- and (**B**) 8-weeks of age and (**C**) *Wt1*^*+/R394W*^ animals at 4 weeks and (**D**) 8 weeks, euthanized at 24 hours, 3 days and 7 days post injection. The cytoskeletal marker synaptopodin (green) was used to discern podocytes. Scale bars = 50 μm. (**E**) Graphs showing percentage of mCherry positive glomeruli at 24 hours with diminished expression 3- and 7-days post injection in 4-week and (**F**) 8-week *Wt1*^+/+^ and *Wt1*^*+/R394W*^ animals. Each data point represents the mean of 50 glomeruli/animal (*n =* 3 / group, *n =* 2 for 8-week *Wt1*^*+/R394W*^ animals at 7 days). Data represents mean ± SEM, (two-way ANOVA with Sidak’s multiple comparisons, **P≤0.01, ***P≤0.001).

Mice of each genotype and age were euthanized at 24 hours, 3 days or 7 days post injection and the injected kidneys were stained for mCherry and the podocyte marker synaptopodin. Representative micrographs for all groups showed mCherry positive staining within glomeruli, indicating the efficacy of the RWrNM peptide in localizing nanocomplexes to the glomerulus up to 7 days post injection in *Wt1*^*+/+*^ and *Wt1*^*+/R394W*^ animals at 4- and 8- weeks of age (Fig. 3A to D). At the cellular level, mCherry showed minimal colocalization with synaptopodin, suggesting the primary site of uptake to be gECs (Fig. 3A to D).

The percentage of mCherry positive glomeruli at 24 hours post injection was 75.3% ± 3.71 and 81.3% ± 2.90 in 4-week-old and 66.0% ± 16.8 and 76.3% ± 12.6 in 8-week-old *Wt1*^*+/+*^ and *Wt1*^*+/R394W*^ mice respectively (Fig. 3E and F). For animals at 4 weeks of age, this was significantly reduced to 25.3% ± 4.80 (*Wt1*^+/+^) and 38.7% ± 8.96 (*Wt1*^*+/R394W*^) by 3 days post injection (Fig. 3E; *P* ≤ 0.001 for both genotypes 24 hours versus 3 days post injection). Similarly, in 8-week-old animals the number of mCherry positive glomeruli was reduced by 3 days post injection to 34.7% ± 8.4 (*Wt1*^*+/+*^) and 35.3% ± 19.2 (*Wt1*^*+/R394W*^) compared with 24 hours, although this did not reach significance (Fig. 3F; *P* = 0.30 and *P* = 0.13 for 8-week-old *Wt1*^*+/+*^ and *Wt1*^*+/R394W*^ mice respectively between 24 hours and 3 days). From 3 to 7 days, there was no further reduction in mCherry positive glomeruli, with 40.0% ± 4.2 and 37.7% ± 7.3 for 4-week-old and 38.3% ± 4.2 and 53% ± 5.7 for 8-week-old *Wt1*^*+/+*^ and *Wt1*^*+/R394W*^ mice respectively (Fig.3 E and F; *P* = 0.26 and *P* > 0.99 for 4-week-old and *P* = 0.99 and *P* = 0.79 for 8-week-old *Wt1*^*+/+*^ and *Wt1*^*+/R394W*^ mice respectively between 3 and 7 days).

There was no difference between the percentage of mCherry positive glomeruli in *Wt1*^*+/+*^ and *Wt1*^*+/R394W*^ mice at 24 hours, 3 days or 7 days post injection (fig. S7A and B, *P* = 0.85, *P* = 0.33 and *P* = 0.98 for 4-week old and *P* = 0.93, *P* > 0.99 and *P* = 0.86 for 8-week old *Wt1*^*+/+*^ and *Wt1*^*+/R394W*^ mice at 24 hours, 3 days and 7 days respectively), indicating that disease state does not affect glomerular RWrNM nanocomplex uptake. Additionally, we pooled *Wt1*^*+/+*^ and *Wt1*^*+/R394W*^ animals at each time point post injection and found no significant differences between 4- and 8-week animals (fig. S7C, *P* = 0.83, *P* = 0.98 and *P* = 0.93 for 24 hours, 3 days and 7 days respectively between 4- and 8-week-old animals), suggesting that glomerular uptake is similar across both analysed ages.

### *Angpt1* nanocomplexes are stable for one month and their delivery enhances TIE2 kidney phosphorylation in *Wt1*^*+/R394W*^ kidneys

**Fig. 4:**
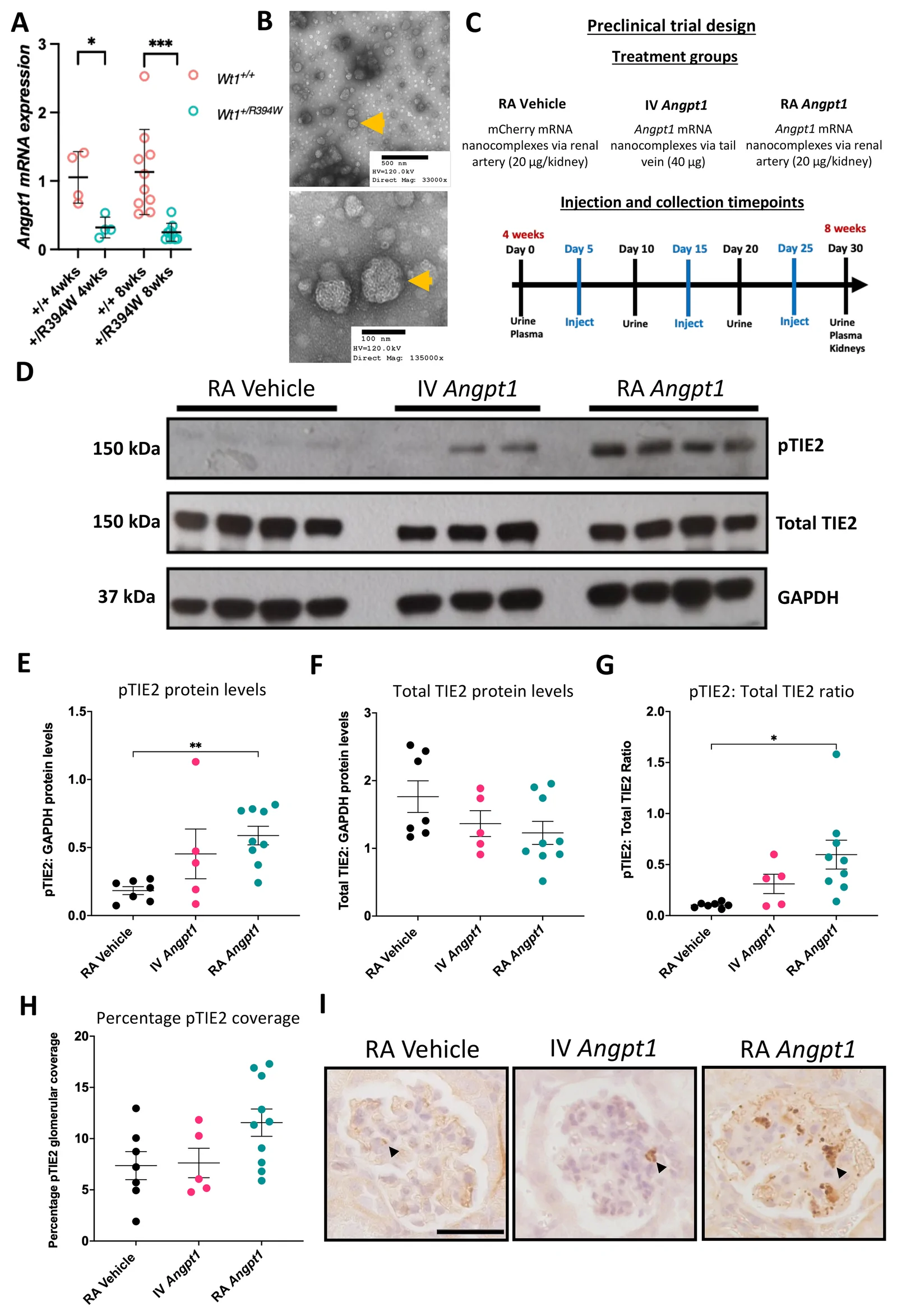
*Angiopoietin-1* mRNA nanocomplexes enhance TIE2 kidney phosphorylation in *Wt1*^*+/R394W*^ mice. (**A**) *Angpt1* mRNA expression in *Wt1*^*+/R394W*^ primary podocytes versus *Wt1*^*+/+*^ at 4- (*n =* 4/group) and 8-weeks of age (*n =* 10 for *Wt1*^*+/+*^ and *n =* 9 for *Wt1*^*+/R394W*^). Each point represents podocytes from one animal. Data represents mean ± SEM (unpaired t-test and Mann-Whitney U test, * *P*≤0.05, ***P≤0.001); *Gapdh* used as the housekeeping gene. (**B**) Negative stain TEM showing spherical nanocomplexes (50 nm – 100 nm size; yellow arrowheads) captured at 3300x (top; scale bar is 500 nm) and 135000x magnification (bottom; scale bar is 100nm). (**C**) Preclinical trial design in 4-week-old *Wt1*^*+/R394W*^ mice comparing RA vehicle, IV *Angpt1* and RA *Angpt1* treatment, with urine, plasma collection, and injection timepoints shown over the 4-week trial. (**D**) Representative Western blots showing kidney cortical pTIE2 (150 kDa), total TIE2 (150 kDa) and GAPDH (37 kDa) from RA vehicle (*n =* 7), IV *Angpt1* (*n =* 5) and RA *Angpt1* (*n =* 9) animals. (**E**) Quantification of pTIE2, (**F**) total TIE2 and (**G**) pTIE2:total TIE2 ratio (one-way ANOVA with Tukey’s multiple comparisons, *P≤ 0.05, **P≤0.01). (**H**) pTIE2 glomerular tuft coverage for RA vehicle (*n* = 7), IV *Angpt1* (*n* = 5) and RA *Angpt1* (*n =* 10); each point = mean of 50 glomeruli/animal. Data represents mean ± SEM (one-way ANOVA with Tukey’s multiple comparisons). (**I**) Representative glomerular micrographs stained for pTIE2 (black arrowheads). Scale bars = 50 μm.

Having demonstrated the efficacy of our nanocomplex formulation in podocytes and gECs in vitro, alongside an effective delivery route facilitating glomerular uptake in vivo, we aimed to assess whether this approach could be used to administer an *Angpt1* RNA therapy. Firstly, we performed CiiiDER analysis, which identified predicted WT1 binding motifs within both the human and mouse *Angpt1* proximal promotor regions, suggesting transcriptional regulation of *Angpt1* by WT1 (fig. S8A). We then assessed *Angpt1* transcript expression in *Wt1*^*+/R394W*^ primary podocytes (cultured ex vivo) and found a significant reduction compared with *Wt1*^*+/+*^ controls at 4- (*P* = 0.011) and 8-weeks of age (*P*≤0.001) (Fig. 4A), akin to findings in mouse models of adult glomerular diseases (14, 18, 19), as well as in patients with CKD (fig. S8B), and corresponding with the loss of gECs observed by 8 weeks of age in *Wt1*^*+/R394W*^ mice (12). Thus, considering the protective role of ANGPT1 on the endothelium (11), we sought to replenish glomerular *Angpt1* expression using mRNA nanocomplexes as a therapeutic strategy for *Wt1*^*+/R394W*^ glomerulopathy.

*Angpt1* nanocomplexes were visualized by negative stain transmission electron microscopy (TEM) (Fig. 4B), displaying spherical particles 50 -100 nm in diameter. Colloidal stability was assessed by measuring hydrodynamic size (fig. S9A) and PDI (fig. S9B) over 28 days at 4°C and 25°C, reflecting clinically accessible storage conditions. At 25°C, nanocomplexes exhibited a 37.9% reduction in mean size (fig. S9A) and a 17.5% rise in PDI (fig. S9B) over 28 days, indicating structural collapse and loss of uniformity. Over the same timeframe, nanocomplexes at 4°C maintained greater integrity and homogeneity, showing a 9.2% reduction in size (fig. S9A) and 9.9% decrease in PDI (fig. S9B). Guided by our previous experiments using 20 μg of luciferase or mCherry mRNA per kidney, pilot studies were performed in which 4-week-old *Wt1*^*+/R394W*^ mutant mice were administered RWrNM nanocomplexes carrying either 10 μg (total 20 μg) or 20 μg (total 40 μg) *Angpt1* into each kidney via the renal artery. Twenty-four hours post injection, animals that received 20 μg of *Angpt1* mRNA per kidney had significantly increased *Angpt1* expression in the kidney cortex, a finding not replicated in animals treated with 10 μg (fig. S10; *P* = 0.052 and *P* = 0.033 for 10 μg and 20 μg respectively versus non injected *Wt1*^*+/R394W*^ animals). Therefore a 20 μg dose per kidney was selected for further studies. Based on our longevity data (Fig. 3), we decided to provide three doses of *Angpt1* nanocomplexes (mean size = 153.6 ± 5.2 nm, fig. S4D; mean PDI = 0.212 ± 0.014; mean ZP = 40.1 ± 0.5 mV; encapsulation efficiency = 97.92 ± 1.77 %) (table S1) into each kidney of *Wt1*^*+/R394W*^ mice between the ages of 4 and 8 weeks.

The design of our interventional preclinical trial consisted of 4-week-old *Wt1*^*+/R394W*^ mice treated with 20 μg mCherry mRNA nanocomplexes delivered via the renal artery of each kidney (RA vehicle), 40 μg *Angpt1* mRNA nanocomplexes delivered via the tail vein (IV *Angpt1*), or 20 μg *Angpt1* mRNA nanocomplexes delivered via the renal artery of each kidney (RA *Angpt1*) (Fig. 4C). To ensure that neither the procedure nor the therapy had a negative effect on overall health, mice were weighed at baseline and five days post each injection. Across all groups weight gradually increased with age (fig. S11), suggesting that therapeutic administration was well tolerated.

At the termination of the experiment at 8 weeks of age, our first aim was to determine the bioactivity of our treatment by assessing the phosphorylation of the ANGPT1 receptor, TIE2 (pTIE2) by Western blot (Fig. 4D). Total protein isolated from the kidney cortex of animals in the IV *Angpt1* group (0.45 ± 0.18) showed pTIE2 expression that was 2.5-fold higher than the RA vehicle group (0.18 ± 0.03), but this did not reach significance (*P* = 0.16). In contrast, the RA *Angpt1* group, had significantly increased expression of pTIE2 (0.59 ± 0.07), 3.3-fold higher than (*P =* 0.01) the RA vehicle group (Fig. 4E). Analysis of total TIE2 in RA *Angpt1* kidney cortex found this significant increase was due to changes in pTIE2 rather than elevated expression of total TIE2, as there was no alteration in total TIE2 protein between any of the groups (Fig. 4F; RA Vehicle 1.76 ± 0.23, IV *Angpt1* 1.37 ± 0.19 and RA *Angpt1* 1.23 ± 0.16, *P* = 0.16). The pTIE2:total TIE2 ratio corroborated this, with the RA *Angpt1* pTIE2:total TIE2 ratio (0.60 ± 0.13) significantly higher than RA Vehicle animals (0.10 ± 0.01), whereas the IV *Angpt1* group did not reach significance (0.31 ± 0.09) (Fig. 4G; *P* = 0.01 and *P* = 0.48 for RA *Angpt1* and IV *Angpt1* versus RA vehicle respectively).

We then examined if pTIE2 was localized within glomeruli using immunohistochemistry. In accord with the Western blot data, there was a 1.6-fold increase in mean percentage pTIE2 glomerular coverage in the RA *Angpt1* group (11.56%) compared with both the RA vehicle (7.36%, *P* = 0.094) and IV *Angpt1* (7.62%, *P* = 0.174) groups, although these increases were not significant over 50 glomeruli analysed/animal (Fig. 4H). Representative micrographs support this trend, with more pTIE2 staining in the glomerular tuft of RA *Angpt1* animals (Fig. 4I).

### *Angpt1* nanocomplex therapy improves glomerular structure and function in *Wt1*^*+/R394W*^ mice

**Fig. 5:**
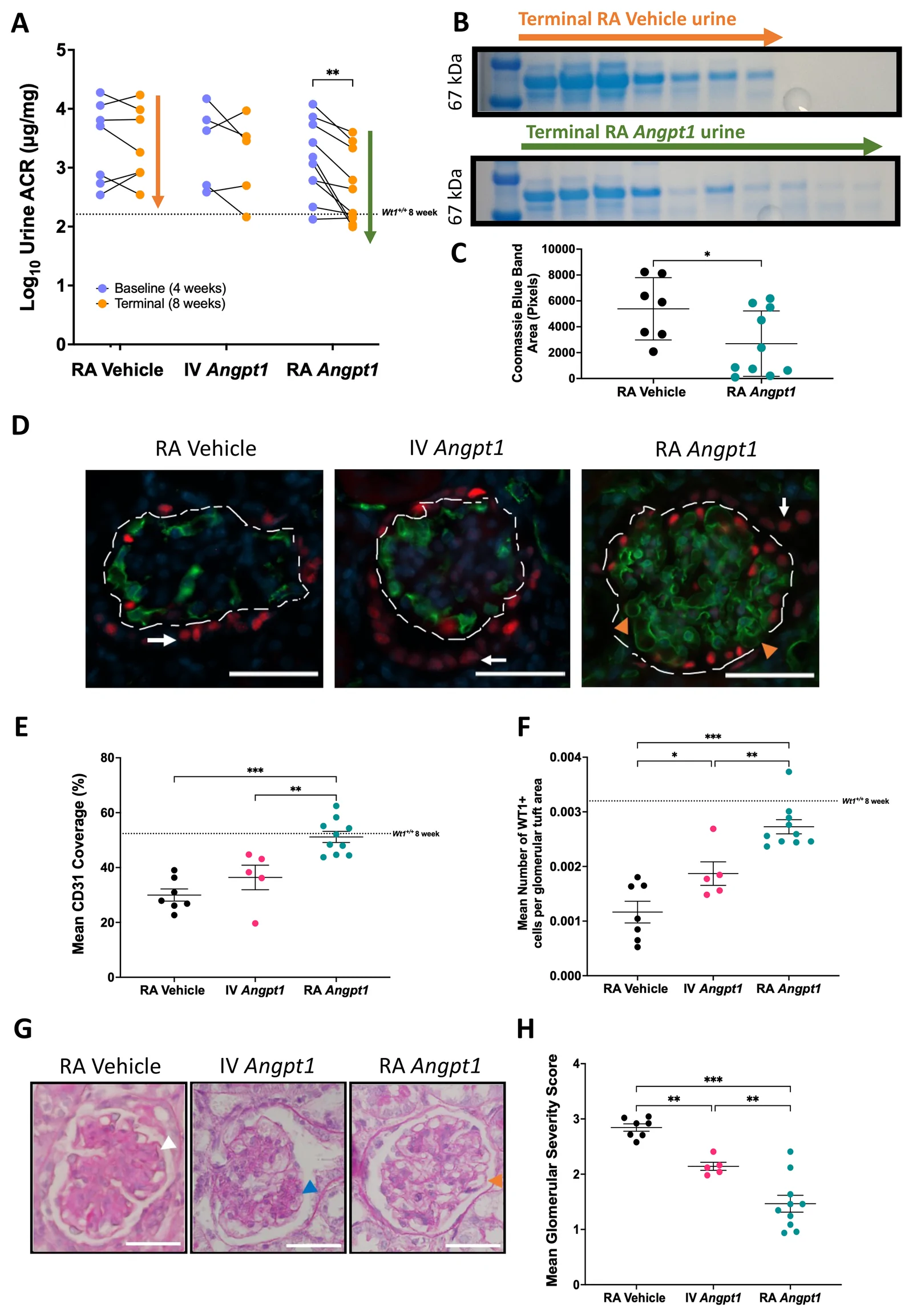
*Angiopoietin-1* mRNA nanocomplexes alleviate glomerular disease in *Wt1*^*+/R394W*^ mice. (**A**) Log_10_ urinary ACR at baseline and termination for RA Vehicle (*n = 7*), IV *Angpt1* (*n = 5*), and RA *Angpt1* (*n = 9*; one excluded) treated *Wt1*^*+/R394W*^ mice. Each point = one animal; linked pairs (Two-way matched ANOVA, Sidak’s test, **P ≤ 0.01). (**B**) Coomassie SDS-PAGE of terminal urine showing albumin (67 kDa) in RA *Angpt1* (*n* = 10) vs RA Vehicle (*n* = 7). (**C**) Albumin band densitometry from panel B (mean ± SEM; Mann-Whitney U test, *P≤ 0.05). (**D**) Glomerular CD31 (green) and WT1 (red) staining (RA Vehicle, n = *7*; IV *Angpt1*, n = *5*; RA *Angpt1, n* = 10). Dotted line = tuft, white arrows = WT1^+^ cells outside tuft, orange arrowheads = capillaries. (**E**) CD31^+^ tuft coverage and (**F**) WT1^+^ cells/tuft area; dashed line at mean value from our historical *Wt1*^+/+^ animals (12). (**G**) PAS-stained glomeruli with scarring (white), mild-moderate deposition (blue), and capillary loops (orange). (**H**) Glomerular severity scores. Each point = mean of 50 glomeruli/animal. Data are mean ± SEM (one-way ANOVA, Tukey’s test, *P ≤ 0.05, **P ≤ 0.01, ***P ≤ 0.001). Scale bars = 50 μm.

We then examined the glomerular structure and function of *Wt1*^*+/R394W*^ mice following *Angpt1* RNA therapy. As a measure of glomerular function, urinary albumin:creatine ratio (ACR) was monitored throughout the treatment period and showed an average significant decline of 21.4% from 4 to 8 weeks of age in RA *Angpt1* animals (Fig. 5A; log_10_ ACR of RA *Angpt1* 4 week 3.14 ± 0.20 versus RA *Angpt1* 8 week 2.64 ± 0.17 μg/mg, *P =* 0.0018). The ACR in the RA *Angpt1* was reduced to values comparable to that observed in our historical *Wt1*^+/+^ measurements at 8 weeks of age (log_10_ 2.21 ± 0.12 μg/mg). In contrast, there was no change in ACR from 4 to 8 weeks in both RA vehicle (4 week 3.43 ± 0.23 versus 8 week 3.38 ± 0.22 μg/mg; *P =* 0.98) and IV *Angpt1* treatment groups (4 week 3.38 ± 0.27 versus 8 week 3.16 ± 0.30 μg/mg; *P =* 0.52). Coomassie blue staining was also performed on terminal urine samples from the RA Vehicle and RA *Angpt1* groups (Fig. 5B). The RA *Angpt1* treated group exhibited bands with significantly smaller pixel area at 67 KDa, the size of albumin, compared with RA Vehicle mice (Fig. 5C; *P* = 0.04). As an overall indication of kidney health, BUN was measured at 4 and 8 weeks of age (fig. S12A). Paired analysis showed no significant change in BUN for any group (RA vehicle, *P =* 0.99; IV *Angpt1, P =* 0.99; RA *Angpt1, P =* 0.57). Plasma creatinine was assessed in terminal blood plasma at 8 weeks of age by mass spectrometry and showed no significant difference between any group (fig. S12B; *P* = 0.19).

Due to the anti-apoptotic, anti-inflammatory, and stabilising effect of ANGPT1 on endothelial cells (14, 18) and the significant loss of gECs observed in *Wt1*^*+/R394W*^ mice (12) by 8 weeks, we next assessed gEC coverage across treatment groups at 8 weeks of age using CD31 immunostaining (Fig. 5D). We found that glomerular CD31^+^ percentage coverage was significantly increased in the RA *Angpt1* animals (51.2% ± 2.3) compared with either RA vehicle (29.9% ± 2.2) (Fig. 5E; *P* < 0.001) or IV *Angpt1* (36.4% ± 4.3) groups (*P =* 0.004), a percentage similar to that seen in our historical samples of *Wt1*^*+/+*^ animals (52.39 ± 4.0% (12)). In contrast, there was no significant difference between RA vehicle and IV *Angpt1* groups (*P =* 0.45).

To determine if *Angpt1* therapy was impacting podocytes we quantified the number of WT1^+^ cells within the glomerular tuft area. In the RA *Angpt1* group, we found a significant increase in the number of WT1^+^ cells per tuft area (2.7 × 10^-3^ ± 1.3 × 10^-4^) compared with RA vehicle (1.2 × 10^-3^ ± 1.8 × 10^-4^; *P <* 0.001), and IV *Angpt1* (1.9 × 10^-3^ ± 2.1 × 10^-4^; *P* = 0.009) groups (Fig. 5D and F), a number similar to that observed in our historical *Wt1*^*+/+*^ animals (3.2 × 10^-3^ ± 3.9 × 10^-4^ (12)). A significantly higher number of WT1^+^ cells was also observed in the IV *Angpt1* group compared to RA vehicle group (*P =* 0.002).

One of the pathological hallmarks of WT1 glomerulopathy is glomerulosclerosis, the deposition of ECM components in the mesangium which leads to irreversible glomerular scarring, reducing its filtration capacity. In the *Wt1*^*+/R394W*^ model, similar to gEC loss, glomerulosclerosis is present by 8 weeks of age, defining an irreversible stage of disease (12). Therefore, we assessed glomerulosclerosis, measured by a pathological severity score index (32), to determine whether RA *Angpt1* therapy resulted in reduced scarring. Representative micrographs of the RA vehicle group (Fig. 5G) show matrix deposition and sclerosis, with few capillary loop structures. In IV *Angpt1* animals, glomeruli showed more open capillary loop structures and less sclerosis, but with some regions of matrix deposition (Fig. 5G). This matrix deposition was visibly reduced in RA *Angpt1* glomeruli, with an increase in the number of visible open capillary loops (Fig. 5G). Blinded quantitative analysis showed RA *Angpt1* animals had a significantly lower glomerulosclerosis score (1.47 ± 0.14) than either RA vehicle (2.84 ± 0.07; *P <* 0.001) or IV *Angpt1* (2.14 ± 0.07; *P* = 0.007) groups (Fig. 5H). Similar to that observed with podocyte number, IV *Angpt1* treatment improved glomerulosclerosis compared with the RA vehicle group (Fig. 5H, *P* = 0.008).

Collectively, these clinically-relevant metrics showed a significant improvement in ACR, rescued gEC and podocyte number, and prevention of glomerulosclerosis after three doses of *Angpt1* RWrNM nanocomplex therapy via renal artery administration in *Wt1*^*+/R394W*^ mice. In contrast, only partial prevention of glomerulosclerosis and podocyte number was achieved through IV administration, highlighting the importance of local kidney delivery.

## Discussion

Our study describes a glomerular-targeted *Angpt1* nanocomplex that, when delivered through the renal artery, slows disease progression in a genetically orthologous WT1 glomerulopathy model. This work establishes a targeted RNA therapeutic approach for a congenital glomerular disorder lacking effective treatments and provides a foundation for clinical translation to improve outcomes in affected children.

To localize our *Angpt1* therapy to the glomerulus we adopted a lipid nanocomplex approach allowing for small peptide targeting to integrin αvβ3. We used the targeting motif RWrNM to increase uptake by two key cells implicated in WT1 glomerulopathy, podocytes and gECs (12). Compared with cyclic RGD, RWrNM improved transfection efficiency of both cell types in vitro, aligning with findings in tumour cells (31) and rat primary podocytes (33). This is the first use of the RWrNM peptide in combination with cationic lipid nanocomplexes (23) to deliver nucleic acids, providing a strong foundation for future RNA therapies.

Systemic administration for renal therapies is limited by reticuloendothelial system (RES)-mediated clearance, causing high liver and spleen accumulation (34). Consistent with this, we found no kidney localization of luciferase RWrNM nanocomplexes following IV injection, in accord with a prior study using integrin αvβ3-targeted RWrNM micelles (33), underscoring the need for alternative delivery routes even for glomerular-targeted vehicles. Renal artery injection bypasses the RES, improving kidney uptake, highlighted by microbubble (35), microcapsule (36), extracellular vesicle (37) and stem cell (38) delivery. However, these studies utilise surgical approaches like aortic ligation (37) or catheter-based delivery (36), posing risks such as hematoma (39). By contrast, ultrasound-guided renal artery injection is minimally invasive, requires no surgery (20) and achieves approximately 80% kidney-specific localization of luciferase nanocomplexes.

Our non-viral strategy facilitated glomerular retention of nanocomplexes in wild-type and diseased mice, consistent with findings in other murine glomerular disease models (40). This contrasts with smaller AAVs, which are detected in the urine of mice (41) and non-human primates (42) post IV injection, with a further report showing a 10^4^ fold increase in urinary AAV9 concentration in CKD mice compared with wildtype controls, a finding associated with increased glomerular permeability (43). The absence of colocalization between mCherry and the podocyte marker synaptopodin suggests that our nanocomplexes primarily interact with gECs, likely reflecting their larger size compared with other delivery vectors, reducing the chances of urinary loss. In this context, gEC localization is not expected to limit therapeutic efficacy as ANGPT1 is a secreted molecule that acts locally to maintain gEC health (11). Moreover, *Angpt1* nanocomplexes were smaller than the mCherry nanocomplexes used for cell localization analysis, increasing the likelihood of podocyte uptake and potential enhancement of natural podocyte-gEC crosstalk. Overall, local provision to the glomerulus is sufficient for ANGPT1 to elicit a protective effect in *Wt1*^*+/R394W*^ mice.

Podocyte overexpression of *Angpt1* in adult diabetic mice also reduces albuminuria (14) and prevents gEC proliferation. Although providing a strong proof of concept for *Angpt1* therapy, this transgenic approach is not clinically feasible. In a more translational strategy, IV delivered COMP-ANGPT1 adenoviruses also reduced albuminuria in diabetic mice, alongside attenuation of mesangial matrix deposition and GBM thickening, slowing progression of glomerulosclerosis (44). Despite using an untargeted vector and systemic delivery, the efficacy of this approach may reflect the use of COMP-ANGPT1, a more potent ANGPT1 variant, with enhanced TIE2 activation. Our data adds to these observations, showing that *Angpt1* therapy is also protective in congenital glomerular disease, improving albuminuria and preventing glomerulosclerosis. *Angpt1* expression is also reduced in rodent models of glomerulonephritis (18), and focal segmental glomerulosclerosis (19)colloidal stability and reduced aggregation. Additionally, we report *ANGPT1* is decreased in human CKD, a finding confirmed by others (45). Furthermore, an ANGPT1 mimic is protective in a unilateral urethral obstruction murine model of CKD (45). Therefore, our nanocomplex platform, which has the advantage of being inherently scalable to treat large populations and enables glomerular targeting of *Angpt1*, may have broader therapeutic applications in acquired glomerular diseases and in the wider context of CKD.

RA *Angpt1*-treated mice had higher gEC coverage and reduced vascular permeability compared with RA vehicle and IV *Angpt1* groups. This aligns with the known protective effects of ANGPT1, shown to enhance endothelial cell survival (46), reduce vascular hydraulic conductivity and increase albumin retention (16), as well as improve peri-vascular cell (17) and endothelial glycocalyx (16) coverage, all of which act to prevent vascular leakage (47). In the kidney, *Angpt1* overexpression prevents microvascular loss in preclinical models of kidney injury induced by either ureteral obstruction (48), ischemia (49) or toxins (50). In the latter study (50), although systemic *Angpt1* adenoviral delivery prevented microvascular loss in a folic acid induced nephropathy model, this was accompanied by a pro-inflammatory response, likely mediated by TIE2 expression on macrophages and neutrophils (51). This observation highlights the importance of localized *Angpt1* delivery which may limit potential confounding effects seen following systemic administration.

We also found that RA delivered *Angpt1* nanocomplexes increased WT1^+^ cells per tuft area, more than IV *Angpt1* and RA vehicle groups. IV *Angpt1* also increased the number of WT1^+^ cells versus vehicle, suggesting circulating nanocomplexes reached the glomerulus, albeit to a lesser extent. One explanation for this is gEC uptake and secretion of ANGPT1 across the GBM to reach podocytes, also known to express TIE2 (14, 52). Alternatively, smaller *Angpt1* nanocomplexes may traverse gEC fenestrae to reach podocytes directly. DLS showed that *Angpt1* nanocomplexes were, on average, 73.8 nm smaller than mCherry complexes, likely because *Angpt1* mRNA was prepared in water, whereas mCherry used citrate buffer, which can promote salt formation and aggregation (53). TEM confirmed the presence of smaller *Angpt1* nanocomplexes ranging from 50 to 100 nm in dry-state. Either way, it is likely that the restoration of either cell type would mediate a positive feedback loop, normalising the cellular crosstalk that is vital for both podocyte and endothelial function (11). Thus, rescued podocyte number could be partially attributed to the improvement in gEC health. In turn, the increased podocyte number may also protect against glomerulosclerosis, as podocytes prevent GBM bulging and subsequent extracellular matrix deposition that leads to scarring (54).

Our therapeutic strategy shows promise for translation into the clinic to treat human WT1 glomerulopathy. Ultrasound-guided injections are part of clinical practice for steroid (55) and anticoagulant (56) delivery. Furthermore, cationic lipid nanoparticles have shown safety worldwide in COVID-19 vaccine recipients (57). Our *Angpt1* nanocomplex formulation also remained stable for up to one month at 4°C, an accessible storage condition that supports wide patient access. This stability is consistent with the high ZP (+40.1 ± 0.5 mV) of our nanocomplexes, as values above +30 mV are associated with strong colloidal stability and reduced aggregation (58). Importantly, treatment was initiated after the onset of increased albuminuria, reflecting a clinically relevant interventional design. We speculate that ANGPT1 therapy could complement existing treatment strategies for congenital glomerular disease, including inhibitors of the renin-angiotensin system, which can modulate ANGPT1 expression (59), to provide a synergistic approach for the management of WT1 glomerulopathy and potentially other glomerular diseases.

This study has several limitations. From our longevity analyses, mCherry protein expression dropped at 3 days post injection and despite modifications to the *Angpt1* mRNA such as codon optimisation (60) and uridine substitution (61), we still reasoned that three therapeutic doses over a 4-week period were required for our preclinical trial. Further work is required to determine the minimal dosing regimen, as repeated dosing, although clinically feasible, may pose challenges for long-term implementation, introducing risks of procedural burden and patient compliance. Despite this, renal arterial access is routinely performed for diagnostic angiography and is used repeatedly in pediatric patients undergoing angioplasty (62). Furthermore, the transient expression of mRNA enabled treatment discontinuation if required, a limitation of viral approaches which can result in sustained, uncontrollable transgene expression (63). In the present study, ultrasound-guided renal artery injection of *Angpt1* nanocomplexes was well tolerated, enabling three administrations over four weeks without procedure-related mortality. A second limitation reflects the use of plasma creatinine and BUN as kidney function markers in mice and the lack of differences observed with *Angpt1* treatment. In mice, creatinine undergoes substantial tubular secretion (64), which can mask reductions in glomerular filtration and thereby confound values until global nephron dysfunction occurs. BUN is influenced by tubular urea reabsorption, as well as systemic urea production (65), limiting its sensitivity and specificity as a marker of early glomerular injury. Thus a longer duration of therapeutic intervention may be required for structural recovery to translate into detectable improvements in plasma creatinine and BUN, and future studies should incorporate additional metrics such as real-time monitoring of FITC-sinistrin clearance or plasma Cystatin C to provide a more comprehensive assessment of kidney function. Further work is also required to understand the molecular pathways by which ANGPT1 exerts its therapeutic effect. Finally, toxicology was not assessed in this current study but is important for future work as mRNA therapies can trigger immunogenicity (66).

In conclusion, we have developed an αvβ3-targeted lipid nanocomplex platform for delivering mRNA to glomeruli, achieving effective transfection in vitro and localized administration in vivo using an ultrasound-guided delivery technique. Using this bespoke technology, we delivered *Angpt1* and demonstrated therapeutic benefit in *Wt1*^*+/R394W*^ mice, reducing albuminuria and glomerulosclerosis severity, as well as rescuing podocyte and gEC loss. Together this work provides a basis for the further clinical development of this therapy, to slow disease progression and to prolong the lives of patients with glomerular disease.

## Materials and Methods

### Study design

This study was designed to test *Angpt1* lipid nanocomplex therapy in a murine model of WT1 glomerulopathy. Lipid nanocomplexes with integrin αvβ3-targeting peptides were tested for uptake in primary murine podocytes and gECs in vitro using luciferase assays (mean of six replicates per group: three independent experiments). In vivo biodistribution and longevity of expression experiments with RWrNM nanocomplexes were carried out with at least two biological replicates. Specific sample numbers for each experiment are detailed in the figure legends. The nanocomplexes used self-assemble on mixing of three components: nucleic acids (mRNA), liposomes and targeting peptides in a 1:3:4 weight ratio.

All animal experiments and procedures were approved by the U.K. Home Office in accordance with the Animals (Scientific Procedures) Act 1986. In our preclinical therapeutic trial, animals were administered either via the tail vein or renal artery under ultrasound-guidance with three doses of *Angpt1* mRNA (encapsulated in RWrNM nanocomplexes) from 4 to 8 weeks of age. mCherry mRNA was used as a vehicle control. Sample size was determined by power calculations based on previous data (12) of log ACR in *Wt1*^*+/R394W*^ mice at 8 weeks of age (*n = 18*), the mean of which was 3.57 with a standard deviation of 0.69. We sought to achieve a 30% decrease in line with UK Renal Association guidelines, which would reclassify a patient with severely increased ACR (category A3; at 30 mg/mmol) down to moderately increased (A2; at 21 mg/mmol); at 80% power, 0.05 two-tailed significance, this required a minimum *n* of *7*. Animals were assigned randomly to each experimental group, monitored and weighed throughout the experiment. Our primary outcome, urinary ACR, was measured every 10 days from 4 to 8 weeks of age. Blood was collected at 4 weeks and 8 weeks of age to assess BUN and plasma creatinine. Tissues were harvested at 8 weeks and processed for Western blot, histology, and immunofluorescence. Animals were excluded from a specific analysis if there was insufficient urine, plasma, or tissue. Glomerulosclerosis scoring was conducted blinded and all other analyses were not blinded

### Statistical analysis

Data are presented as mean ± standard error of mean (SEM), unless otherwise indicated and analysed using GraphPad Prism v10. Normality was assessed by Shapiro-Wilk test. When comparing 2 groups, data were analysed using an unpaired t-test (normally distributed) or a Mann-Whitney U test (not normally distributed). When comparing 3 or more groups, a one-way ANOVA with Tukey’s multiple comparison (normally distributed) was used. When comparing paired data, i.e., from the same biological entity at two different time points, a paired t-test was carried out for 2 groups and a 2-way matched ANOVA, with Sidak’s multiple comparisons, for 3 or more groups. *P ≤ 0.05, **P ≤ 0.01, ***P ≤ 0.001 was considered significant. All individual level data for *n* < 20 are presented in data file S1.

## Supplementary Material

Supplementary Checklist

Supplementary Data File S1

Supplementary materials

## Data Availability

All data associated with this study are present in the paper or Supplementary Materials. Requests for lipid nanocomplexes should be addressed to DAL under a material transfer agreement.

## Overline

**One sentence summary**

*Angiopoietin-1* RNA therapy slows the progression of WT1 glomerular disease in a preclinical mouse model.
