# Supplementary material for: Ultrasound-guided renal artery administration of angiopoietin-1 RNA therapy slows the progression of preclinical WT1 glomerular disease
Source: Sci Transl Med. Author manuscript; Available in PMC 2026 Oct 10. (PMC7619540; doi:10.1126/scitranslmed.adv1289)
Supplement: Supplementary file 1 [file EMS218099-supplement-Supplementary_Checklist.pdf]

Materials Design Analysis Reporting (MDAR)
Checklist for Authors

The MDAR framework establishes a minimum set of requirements in transparent reporting applicable to studies in the life sciences (see Statement of Task: doi:10.31222/osf.io/9sm4x.). The MDAR checklist is a tool for authors, editors, and others seeking to adopt the MDAR framework for transparent reporting in manuscripts and other outputs. Please refer to the MDAR Elaboration Document for additional context for the MDAR framework.

For all that apply, please note where in the manuscript the required information is provided.

Materials:

Newly created materials	indicate where provided: page no/section/legend)	n/a
The manuscript includes a dedicated "materials availability statement" providing transparent disclosure about availability of newly created materials including details on how materials can be accessed and describing any restrictions on access.	Acknowledgments	

Antibodies	indicate where provided: page no/section/legend)	n/a
For commercial reagents, provide supplier name, catalogue number and RRID, if available.	Materials and methods > Immunofluorescence<br>Materials and methods > Immunohistochemistry<br>Materials and methods > Protein expression by Western blotting	

DNA and RNA sequences	indicate where provided: page no/section/legend)	n/a
**Short novel DNA or RNA including primers, probes:** Sequences should be included or deposited in a public repository.	Materials and methods > Real-time quantative PCR	

Cell materials	indicate where provided: page no/section/legend	n/a
**Cell lines:** Provide species information, strain. Provide accession number in repository **OR** supplier name, catalog number, clone number, **OR** RRID.		n/a
**Primary cultures:** Provide species, strain, sex of origin, genetic modification status.	Materials and methods > Cell culture and in vitro transfections	

Experimental animals	indicate where provided: page no/section/legend)	n/a
**Laboratory animals or Model organisms:** Provide species, strain, sex, age, genetic modification status. Provide accession number in repository **OR** supplier name, catalog number, clone number, **OR** RRID.	Materials nd methods > Animals	
**Animal observed in or captured from the field:** Provide species, sex, and age where possible.		n/a

Plants and microbes	indicate where provided: page no/section/legend)	n/a
**Plants:** provide species and strain, ecotype and cultivar where relevant, unique accession number if available, and source (including location for collected wild specimens).		n/a
**Microbes:** provide species and strain, unique accession number if available, and source.		n/a

Human research participants	indicate where provided: page no/section/legend) or state if these demographics were not collected	n/a
If collected and within the bounds of privacy constraints report on age, sex and gender or ethnicity for all study participants.	Materials and methods > Human tissue	

Design:

Study protocol	indicate where provided: page no/section/legend)	n/a
If study protocol has been pre-registered, provide DOI. For clinical trials, provide the trial registration number **OR** cite DOI.		n/a

Laboratory protocol	indicate where provided: page no/section/legend)	n/a
Provide DOI **OR** other citation details if detailed step-by-step protocols are available.	Materials and methods > Cell culture and *in vitro* transfections	

Experimental study design (statistics details)		
**For in vivo studies:** State whether and how the following have been done	**indicate where provided: page no/section/legend. If it could have been done, but was not, write not done**	**n/a**
Sample size determination	Materials and methods > Study Design	
Randomisation	Materials and methods > Study Design	
Blinding	Materials and methods > Study Design	
Inclusion/exclusion criteria	Materials and methods > Study Design	

Sample definition and in-laboratory replication	indicate where provided: page no/section/legend	n/a
State number of times the experiment was replicated in laboratory.	Materials and methods > Study Design	
Define whether data describe technical or biological replicates.	Materials and methods > Study Design	

Ethics	indicate where provided: page no/section/legend	n/a
**Studies involving human participants:** State details of authority granting ethics approval (IRB or equivalent committee(s), provide reference number for approval.	Materials and methods > Human tissue	
**Studies involving experimental animals:** State details of authority granting ethics approval (IRB or equivalent committee(s), provide reference number for approval.	Materials and methods > Animals	
**Studies involving specimen and field samples:** State if relevant permits obtained, provide details of authority approving study; if none were required, explain why.		n/a

Dual Use Research of Concern (DURC)	indicate where provided: page no/section/legend	n/a
If study is subject to dual use research of concern regulations, state the authority granting approval and reference number for the regulatory approval.		n/a

Analysis:

Attrition	indicate where provided: page no/section/legend	n/a
Describe whether exclusion criteria were preestablished. Report if sample or data points were omitted from analysis. If yes report if this was due to attrition or intentional exclusion and provide justification.	Materials and methods > Study Design	

Statistics	indicate where provided: page no/section/legend	n/a
Describe statistical tests used and justify choice of tests.	Materials and methods > Statistical analyses	

Data availability	indicate where provided: page no/section/legend	n/a
For newly created and reused datasets, the manuscript includes a data availability statement that provides details for access or notes restrictions on access.	Acknowledgements > Data and materials availability	
If newly created datasets are publicly available, provide accession number in repository **OR** DOI **OR** URL and licensing details where available.		n/a
If reused data is publicly available provide accession number in repository **OR** DOI **OR** URL, **OR** citation.		n/a

Code availability	indicate where provided: page no/section/legend	n/a
For all newly generated custom computer code/software/mathematical algorithm or re-used code essential for replicating the main findings of the study, the manuscript includes a data availability statement that provides details for access or notes restrictions.		n/a
If newly generated code is publicly available, provide accession number in repository, **OR** DOI **OR** URL and licensing details where available. State any restrictions on code availability or accessibility.		n/a
If reused code is publicly available provide accession number in repository **OR** DOI **OR** URL, **OR** citation.		n/a

Reporting

MDAR framework recommends adoption of discipline-specific guidelines, established and endorsed through community initiatives. Journals have their own policy about requiring specific guidelines and recommendations to complement MDAR.

Adherence to community standards	indicate where provided: page no/section/legend	n/a
State if relevant guidelines (e.g., ICMJE, MIBBI, ARRIVE) have been followed, and whether a checklist (e.g., CONSORT, PRISMA, ARRIVE) is provided with the manuscript.	Materials and methods > Animals	

Clinical Studies

Adherence to clinical standards	indicate where provided: page no/section/legend	n/a
State if relevant checklists are provided in the supplementary materials e.g., CONSORT for randomized controlled clinical trials, TREND for non randomized studies, REMARK for tumor prognostic studies, BRISQ for human biospecimens. Confirm that a CONSORT flow diagram is in Figure 1		n/a

Clinical Trial Registration	indicate where provided: page no/section/legend	n/a
Confirm that the clinical trial was pre-registered (before patient enrollment) and provide clinical trial registration number from ClinicalTrials.gov or other national registry		n/a

Ethics	indicate where provided: page no/section/legend	n/a
Confirm that informed consent was obtained from all human participants and state the details of the IRB or other authority granting ethics approval including approval reference number		n/a

Demographics Reporting	indicate where provided: page no/section/legend	n/a
Study should follow authoritative standards for reporting demographics data. Descriptors for any demographic identities should be clear, unbiased, and up-to-date.		n/a

Identifiable images	indicate where provided: page no/section/legend	n/a
Authors should remove information from patient photographs that could be used to identify the patient. Where this is not possible, a written release from the patient must be provided.		n/a

Data Access	indicate where provided: page no/section/legend	n/a
Confirm that de-identified human data collected for the study will be made available to researchers/clinicians after publication. Confirm that the manuscript includes a Data and Materials Availability statement that provides details regarding data access or states restrictions on data access. If		n/a

access to data is subject to a data transfer agreement (DTA) or data use agreement (DUA) provide a link to DTA/DUA or provide a copy of the DTA/DUA (which may be included as a supplementary materials file). If there are restrictions on data access, provide a statement regarding which data are restricted and the nature of the restriction.		
