# Supplementary material for: Ultrasound-guided renal artery administration of angiopoietin-1 RNA therapy slows the progression of preclinical WT1 glomerular disease
Source: Sci Transl Med. Author manuscript; Available in PMC 2026 Oct 10. (PMC7619540; doi:10.1126/scitranslmed.adv1289)
Supplement: Supplementary file 2 [file EMS218099-supplement-Supplementary_materials.pdf]

Supplementary materials

Materials and Methods

mRNA lipid nanocomplex formulation

The nanocomplexes used in this study self-assemble on mixing of three components: nucleic acids (mRNA), liposomes and targeting peptides *(23)*. Liposomes were formed by microfluidic mixing of cationic lipids and helper phospholipids. The stock lipids were dissolved in ethanol at a concentration of 50 mg/ml by sonication in an ultrasonic bath for twenty minutes. The sonicated helper and cationic lipid were then mixed in a 1:1 molar ratio in 100% ethanol to generate a total volume of 500 µl. A preparation volume of 2 ml was then achieved by microfluidic mixing of 1.5 ml of ddH2O and 500 µl DOTMA-DOPE in ethanol using a NanoAssemblr (Precision Nanosystems) at a flow rate of 12 ml/min. The resulting liposomes were dialysed for 24 hours by gently spinning a Maxi GeBaFlex-tube (8kDa molecular weight cut-off) (Gene Bio-Applications) in 3 litres of ddH2O to remove ethanol. Liposomes were reconcentrated to 2 mg/ml, sonicated for twenty minutes to reduce liposome size and stored at 4°C.

The cationic lipids tested in this study are shown in **table S2** (Avanti Polar Lipids). The helper phospholipid used is 1,2-dioleoyl-sn-glycero-3-phosphoethanolamine (DOPE). The formulation and ratios described below have been optimised for delivery to multiple cell types including melanoma, colon cancer and lung bronchoalveolar cells *(23)*.

In vitro transfection assay formulations

For in vitro transfections, nanocomplexes comprised of luciferase mRNA (100 ng/ml, TriLink, catalog no. L-7602, diluted in citrate buffer), cationic liposomes (1 µg/µl, diluted in water), and peptide (1 µg/µl, AMSBIO, diluted in water) in a weight ratio of 1:3:4 diluted in Opti-MEM. To target integrin αvβ3, the peptides used were cyclic K_{16}-RVRRGACRGDCLG (cyclic RGD motif), linear K_{16}-GARWrNM (RWrNM motif) and linear K_{16}-GARWRNM (RWRNM, to test the importance of the central arginine chirality (D- or L- Arginine)). The 16-chain poly-lysine (K_{16}) tail on the peptide creates

the correct electrostatics to embed into the liposome, stabilising the negatively charged nucleic acid cargo *(30)*. The RVRR and GA portions of the peptide are cleavable (by the endosomal enzymes cathepsin B and furin) and non-cleavable spacers respectively, important for cellular penetration of the peptide increasing uptake *(30)*.

In vivo transfection assay formulations

For in vivo studies, luciferase (TriLink, catalog no. L-7202) and mCherry (TriLink, catalog no. L-7203, diluted in citrate buffer) mRNAs were used with a 5-methoxyuridine (5moU) modification to improve stability. The *Angpt1* mRNA was bespoke designed, coding for murine *Angpt1* (TriLink), with modifications to increase protein expression and dissolved in water. Unless stated otherwise, for each kidney injection, 20 µg of mRNA was encapsulated into nanocomplexes with 60 µg of C18 DOTMA: DOPE liposomes and 80 µg of RWrNM peptide (1:3:4 ratio) in water. The complexes were left to assemble for 30 minutes before adding 10 µl of 50% dextrose to give a final concentration of 5% dextrose in a total volume of 100 µl. The nanocomplexes were stored at 4 °C until use, never exceeding 4 hours from formulation to in vivo injection.

Nanocomplex characterisation

Nanocomplex size, zeta potential and polydispersity index (PDI) were determined by dynamic light scattering using a Malvern Nano ZS Zetasizer (Malvern Instruments). Sixteen measurements of each formulation mixture were taken for both size and zeta potential, the mean of which was recorded for three independent experiments. PDI is the square of the standard deviation divided by the mean particle diameter and values range from 0 to 1, where 0 and 1 are absolute homogeneity and heterogeneity in nanocomplex size respectively. Size measurements with PDI values of less than 0.3 were accepted as homogenous samples *(67, 68)*. The encapsulation efficiency of mRNA was quantified by Quant-it RiboGreen RNA Assay in accordance with the manufacturer's instructions (Thermo Fisher).

Colloidal stability

Angpt1 mRNA nanocomplexes with RWrNM targeting peptide were formulated as described in the in vitro transfection assay formulations section and stored at 4 °C or 25 °C for one, three, seven, fourteen, twenty-one and twenty-eight days. Formulations were made in parallel for each temperature and time point and after their designated storage conditions, size and PDI were determined. Sixteen measurements for each formulation were taken for size, the mean of which was recorded for three independent experiments.

Transmission electron microscopy

Morphology of the nanocomplexes was evaluated using transmission electron microscopy (TEM). A 5 µl drop of the sample (1 µg/µl lipid concentration) was loaded onto a carbon-coated 300-mesh copper grid (Agar Scientific Ltd) and allowed to stand for three minutes. Excess fluid was then removed with filter paper. For negative staining, the sample placed on the grid was treated with 2% uranyl acetate for three minutes. After removing the excess fluid with filter paper, the grids were dried in air before imaging. TEM images were acquired at an accelerating voltage of 120 kV using a Phillips/FEI CM 120 BioTwin TEM.

Cell culture and in vitro transfections

Primary murine podocytes were isolated from five wild-type $\mathit{Wt1}^{+/+}$ littermates and four mutant heterozygous $\mathit{Wt1}^{+/R394W}$ male mice at 8 weeks of age using magnetic Dynabeads (Invitrogen, catalog no. 14011) as previously described *(12)*. Primary podocytes were plated on Matrigel (10 µl/ml) (Corning) and cultured in RPMI-1640 + 10% Fetal Bovine Serum (FBS), 1% penicillin-streptomycin (P/S), 1% L-Glutamine and 1% insulin-transferrin-selenium.

Primary gECs isolated from C57BL/6 mice were purchased from Cell Biologics (catalog no. C57-6014G), plated in Matrigel coated flasks and grown in complete mouse endothelial cell medium (Cell Biologics, catalog no. M1168), supplemented with 0.1% vascular endothelial growth factor (VEGF), 0.1% endothelial cell growth supplement (ECGS), 0.1% Heparin, 0.1% endothelial growth factor

(EGF), 0.1% Hydrocortisone, 1% Antibiotic-Antimycotic solution and 5% FBS. For both podocytes and gECs, the primary cells were maintained at 37 °C in a 5% CO_2 humidified atmosphere to allow for proliferation and not passaged more than five times.

For in vitro transfections, cells were transfected with luciferase mRNA complexes at 70% confluency. In preliminary experiments, the length of the cationic lipid was optimised (**table S2**) by comparing formulation transfection efficiency in $Wt1^{+/+}$ primary podocytes, complexed to both RGD and RWrNM peptides. To quantify transfection, a luciferase assay (Promega) was carried out according to manufacturer's protocol, 24 hours post transfection. Luminescence was expressed as relative light units per milligram of protein (RLU/mg of protein); the mean value of 6 replicates for each group was determined. For all subsequent experiments, we used the optimal C18 DOTMA: DOPE liposome formulation.

Next, the optimal peptide was determined by luciferase assay with nanocomplex formulations complexed with cyclic RGD, RWrNM and RWRNM in primary murine podocytes and gECs. Control groups included formulations with no mRNA and naked mRNA only. The optimal RWrNM formulation was then directly compared between $Wt1^{+/+}$ and $Wt1^{+/R394W}$ primary podocytes to evaluate the effect of disease state on nanocomplex uptake.

Animals

All animal experiments were approved by the Home Office (PPL: PE52D8C09 and PP1776587) and carried out in accordance with the UK Animals Act 1986 and performed according to the ARRIVE guidelines. All mice used in this study were maintained on standard rodent chow (Teklab Diets) with free access to water in a 12-hour light / 12 hours dark environment. Sample sizes used for each experiment are outlined in Figure Legends.

The *Wt1* disease mouse model in this study contains an amino acid substitution from an arginine to a tryptophan at position 394 of the WT1 protein (R394W) and was generated as described *(13)*. To

observe a phenotype, adult male C57BL/6 mice, heterozygous for the p.R394W *Wt1* mutation (*Wt1*$^{+/R394W}$) were provided by Professor David Bates, University of Nottingham (originally sourced from Professor Vicki Huff) and backcrossed with female MF1s (Charles River Laboratories) for two generations. The second-generation backcrossed male offspring, both wildtype (*Wt1*$^{+/+}$) and mutant mice (*Wt1*$^{+/R394W}$), were used for all subsequent experiments. In heterozygote female animals, the progression of disease is slower than what is observed in males, with no disease symptoms displayed until 16 weeks of age, compared to 4 weeks in males *(13)*. Therefore, only male heterozygotes were used in this study as they phenocopy the rapid progression of human disease and are more fitting for preclinical therapeutic development. Male wild-type animals were used as controls. Genotyping was performed using DNA extracted from ear clips as described *(13)*. For our therapeutic pre-clinical trial, baseline bloods were collected at (4 weeks of age) via tail-bleed and terminal blood collection via cardiac puncture, followed by cervical dislocation for tissue harvest. Spot urine samples were collected every 10 days from 4 to 8 weeks age.

Female CD1 nude mice between 4 to 5 weeks of age (Charles River) were used for biodistribution studies.

Ultrasound guided renal artery injection

This method describes an optimised approach from that originally used for the injection of adipose-derived stem cells *(20)*. An ultrasound imaging system (VEVO 3100, FUJIFILM VisualSonics) with an injection platform was used alongside a MX550S VisualSonics probe with a depth of 15 mm and frequency of 55 MHz to visualise the kidney. Anaesthetised mice were positioned either in the left lateral position, if injecting the right kidney or the right lateral position for left kidney injection**.** Ultrasound gel (Anagel) was applied to the hairless region and the kidney was located, colour doppler mode was used to visualise the blood vessels in the kidney and a pulse wave doppler sample gate was used to obtain velocity waveforms to determine arterial and venous flow**.** The needle was inserted into the renal artery by passing through the paravertebral muscle to avoid damage to the surrounding organs. Once the needle was in place 100 μl of the nanocomplex suspension was injected into the renal artery

of mice using a 1 ml syringe. Following withdrawal of the needle, the mice were placed into a heated chamber to recover from anaesthesia and checked for clinical signs of distress every ten to fifteen minutes. Twenty-four hours post injection the mice were further assessed for signs of discomfort and body weight was recorded.

Bioluminescence imaging and micro-computed tomography

In vivo bioluminescence imaging (BLI) was performed 18 hours post renal artery injection using the in vivo Imaging System (IVIS Spectrum, PerkinElmer). Mice were anaesthetised and intraperitoneally injected with 75 mg/kg of luciferase substrate D-luciferin firefly (Promega) in 200 µl of PBS and imaged after 10 minutes to visualise BLI signal. Grey-scale photographs were acquired with a 24 cm field of view and then a bioluminescence image was obtained using a binning resolution factor of 4. For ex vivo BLI, the mice were immediately sacrificed, and the organs rapidly resected to image within 5 minutes of in vivo imaging. Ex vivo images were acquired for the right injected kidney, left non-injected kidney (control), liver and lungs. For image analysis, live image software (Alameda) was used to place a circular region of interest (ROI) over the whole mouse and right kidney (in vivo) to quantify the signal as total flux (photons/s). The percentage signal in the injected kidney was determined by dividing kidney ROI signal by the whole-body signal (total counts). ROIs were also drawn for the ex vivo organs to quantify signal in each organ.

To further characterise the localisation of luciferase mRNA nanocomplexes, multimodality three-dimensional (3D) bioluminescent and micro-computed tomography (micro-CT) imaging was carried out using an IVIS Spectrum and GX2 micro-CT (Perkin Elmer). Mice were anaesthetised and intraperitoneally injected with 75 mg/kg D-luciferin for BLI, alongside an intravenous injection of 100 µl of iodine for micro-CT contrast. The mouse was then placed in a Perkin Elmer mouse imaging shuttle for whole body micro-CT imaging, acquired with a 72 mm field of view and scan time of four minutes. After acquisition, the 3D image was reconstructed by the QuantumGX system acquisition software. The imaging shuttle was then immediately inserted into the IVIS Spectrum and a 3D reconstruction was generated through diffuse luminescence imaging tomography (DLIT). DLIT

requires a photographic image, alongside a structured light image which helps reconstruct the surface mesh of the mouse and bioluminescent images obtained at multiple emission wavelengths ranging from 560 nm - 660 nm to form a sequence. The combination of these three forms of image produces a DLIT 3D reconstruction with a bioluminescent signal. The DLIT 3D reconstruction was then automatically co-registered with the 3D micro-CT scan in Living image software.

Histological and Biochemical Assessment of Experimental Animals

Urine was collected at 4 and 8 weeks. Glomerular function was assed via urinary albumin/creatinine ratio (ACR) via enzyme-linked immunosorbent assay of albumin (Bethyl Laboratories) and creatinine (Cayman Chemical, catalog no. 500701) according to manufacturer's instructions. Blood urea nitrogen (BUN) was quantified in plasma taken at 4 and 8 weeks of age using the QuantiChrom Urea Assay Kit (BioAssay Systems, catalog no. DIUR-100). Additionally, liquid chromatography tandem mass spectrometry *(69)* performed by SpotOn Clinical Diagnostics, London, UK was used to determine plasma creatinine concentrations at 8 weeks of age. Histological analyses were conducted on 5 μm periodic acid–Schiff-stained sections. For this, fifty glomeruli were imaged per animal using a Zeiss Axioplan histological light microscope. Glomeruli were then blindly scored by three independent individuals under the following categories (0 - 4) for magnitude of glomerular injury: 0, normal glomerular structure; 1, increased mesangial matrix deposition and hypercellularity with some loss of capillary loops; 2, increased matrix deposition and focal areas of sclerosis; 3, >50% of glomerulus sclerotic with very few capillary loops; 4, >75% of glomerulus sclerotic and the presence of glomerular epithelial hyperplasia lesions *(32)*. The mean glomerular score for each animal was plotted.

Human tissue

Human tissue was sourced from Great Ormond Street Hospital for Children NHS Foundation Trust, UK, from patients with *WT1* pathogenic variants who received treatment after 2012, under the remit of the IMPORT/UMBRELLA study, for which we hold ethical approval (London Bridge REC 12/LO/0101, IRAS: 62637) or for those who were not registered under the IMPORT/UMBRELLA study, under the remit of the CCLG tissue bank ethics. The female patient (*WT1 c.1180C>T*;

p.Arg394Trp) was diagnosed with Denys-Drash syndrome (DDS), as defined as having Wilms Tumour, nephropathy presenting as persistent proteinuria or overt nephrotic syndrome (MIM#194080), with histologically defined diffuse mesangial sclerosis. Two paediatric control samples were also used.

Immunofluorescence

Kidney tissue was fixed in 4% paraformaldehyde (PFA) and all immunofluorescence staining performed on 8 µm cryosections, using primary antibodies for mCherry (1:100, Thermo Fisher, catalog no. M11217) and synaptopodin (1:100, 2B Scientific, catalog no. 163-004-SY), or CD31 (1:400, Thermo Fisher, catalog no. MA3105) and WT1 (1:50, Abcam, catalog no. ab89901) diluted in CAS block with 10% secondary host serum. Species specific fluorescent secondary antibodies were used at 1:300 and nuclear staining was achieved by incubation with 10 µg/ml Hoechst (Hoechst 33342, Invitrogen, catalog no. H3570). Sections were imaged on a Zeiss Observer 7 fluorescent microscope. For each stain, fifty glomeruli per animal were imaged and all analysis was carried out in Image J. mCherry positive glomeruli were counted and quantified as a percentage of the total of fifty glomeruli. $CD31^+$ percentage coverage was determined by using the threshold tool to determine the area of the tuft stained for CD31 compared to the total tuft area (*12*). $WT1^+$ cells were counted inside the tuft and normalised to tuft area. The mean values for fifty glomeruli were determined for each animal. Kidney tissue was also embedded in paraffin and sectioned at 8 µm. The sections were stained with integrin β3 (1:50, Novus, catalog no. NBP2-67416) and synaptopodin (described above). Secondary antibody and nuclear staining were carried out as described above.

Primary podocytes and gECs were fixed in 4% PFA before being permeabilised in 0.5% PBST (Triton X-100 in PBS). Cells were stained with primary antibodies for integrin β3 (1:50, Novus, catalog no. NBP2-67416) and either nestin (1:100, Novus, catalog no. NB100-1604), WT1 (described above), and synaptopodin (described above) for podocytes or CD31 (1:400, Thermo Fisher, catalog no. MA3105) for gECs, diluted in CAS block with 10% secondary host serum. Species specific fluorescent secondary antibodies were used at 1:200 and nuclear staining and mounting as previously described.

Immunohistochemistry

All immunohistochemistry staining was performed on 5 µm paraffin sections stained for integrin β3 (described above) and pTIE2 (1:100, Bio-techne, catalog no. AF2720). The slides were then incubated with horseradish peroxidase (HRP) horse anti-rabbit ImmPRESS polymer reagent (Vector Laboratories, catalog no. MP-7401-15) and incubated in HRP substrate 3,3'-Diaminobenzadine (DAB). Sections were counterstained with Mayer's haematoxylin (BioRad) for one minute to visualise nuclei and mounted with Histomount and imaged using a Zeiss Axioplan histological microscope (Zeiss).

Protein expression by Western blotting

Total protein was extracted from kidney cortex by homogenisation in radioimmunoprecipitation assay (RIPA) lysis buffer supplemented with 40 µl/ml cOmplete, EDTA-free Protease Inhibitor Cocktail and 10 µl/ml sodium orthovanadate phosphatase inhibitor. Twenty micrograms of protein sample was denatured with 10% β-mercaptoethanol (Sigma) in Laemmli sample buffer (BioRad) and run on a 4 - 15% sodium dodecyl sulfate–polyacrylamide gel electrophoresis (SDS-PAGE) gradient gels. The gels were transferred to polyvinylidene fluoride membranes and blocked in 5% milk, 0.1% bovine serum albumin (BSA), membranes were then incubated with primary antibodies for pTIE2 (1:1000, described above), TIE2 (1:1000, Bio-techne, catalog no. AF762) and GAPDH (1:20,000, Merck, catalog no. MAB374) with corresponding HRP secondaries at 1:5000. The membranes were then incubated with Pierce ECL Western Blotting Substrate (Thermo Scientific) and detected with X-ray film. Densitometry analysis was carried out in ImageJ for pTIE2 and total TIE2 protein expression relative to GAPDH.

Real-time quantitative PCR

Murine $Wt1^{+/+}$ and $Wt1^{+/R394W}$ podocytes were characterised with podocyte specific markers, *Wt1* and *synaptopodin*. RNA was extracted using the RNeasy plus mini kit (Qiagen, catalog no. 74136), according to manufacturer's protocol. To quantify *β3* and *Angpt1* expression, total RNA was extracted from either wild-type whole murine kidney, kidney cortex or primary murine $Wt1^{+/+}$ and $Wt1^{+/R394W}$ podocytes. cDNA was prepared using the gDNA Clear cDNA Synthesis Kit (Bio-Rad, catalog no. 172-5035). Reverse transcription-quantitative polymerase chain reaction (RT-qPCR) was performed in

duplicate using qPCRBIO SyGreen Mix Lo-ROX (PCRBioSystems, catalog no. PB20.11–05) with gene specific primers for *Wt1, synaptopodin, β3* and *Angpt1*, standardised to *Gapdh*. Murine *Wt1* F: GAGAGCCAGCCTACCATCC and R: GGGTCCTCGTGTTTGAAGGAA; Murine synaptopodin F: CTGCATCCGTGGTCAACAG and R: GGGACTCCTATCCGCCATAC; Murine integrin β3 F: CTGGTGTTTACCACTGATGCCAAG and R: TGTTGAGGCAGGTGGCATTGAAGG; Murine *Angpt1* F: GGTCAACAGAATCGCCACTT and R: GGGCAGGTGAACTCCACTAA; and Murine *Gapdh* F: AGGTCGGTGTGAACGATT and R: TCTAGACCATGTAGTTGAGGTCA.

To validate podocyte expression of *Wt1* and *synaptopodin*, PCR was carried out on five representative primary podocyte samples isolated from *Wt1*$^{+/+}$ and *Wt1*$^{+/R394W}$ mice at 8 weeks of age. After amplification 3 µl of loading buffer was added to 15 µl of PCR product and the amplified fragments were detected on a 2% (w/v) agarose gel stained with SYBR Safe by gel electrophoresis performed at 100 V for thirty-five minutes. Bands were visualised on a Gel Doc EZ System and band size was determined by comparing to a 100- bp ladder.

Coomassie blue gel and quantification

Terminal (8-week) urine samples from all RA Vehicle and RA *Angpt1* animals were separated on a 4 - 15% SDS-PAGE gel. The gels were loaded with 1 µl of urine in Laemmli sample buffer and run for one hour at 160 V. The gels were then incubated in Bio-Safe Coomassie Brilliant Blue R-250 (Bio-rad) for one hour to stain for albumin bands which appeared at 67 kDa.

Predicting WT1 binding motifs (Ciiider)

WT1 motif analysis was done using CiiiDER, within 1.5kb base-pairs downstream and 500 base-pairs upstream of the transcriptional start site *(70)*, with stringency set to 0.1 and the WT1 motif (MA1627.1) defined by JASPAR 2022.

Nephroseq ANGPT1

The Nephroseq database nephroseq.org. was searched for targeted screening of *ANGPT1* and *ANGPT2* differential expression in human datasets.

Supplementary Figures

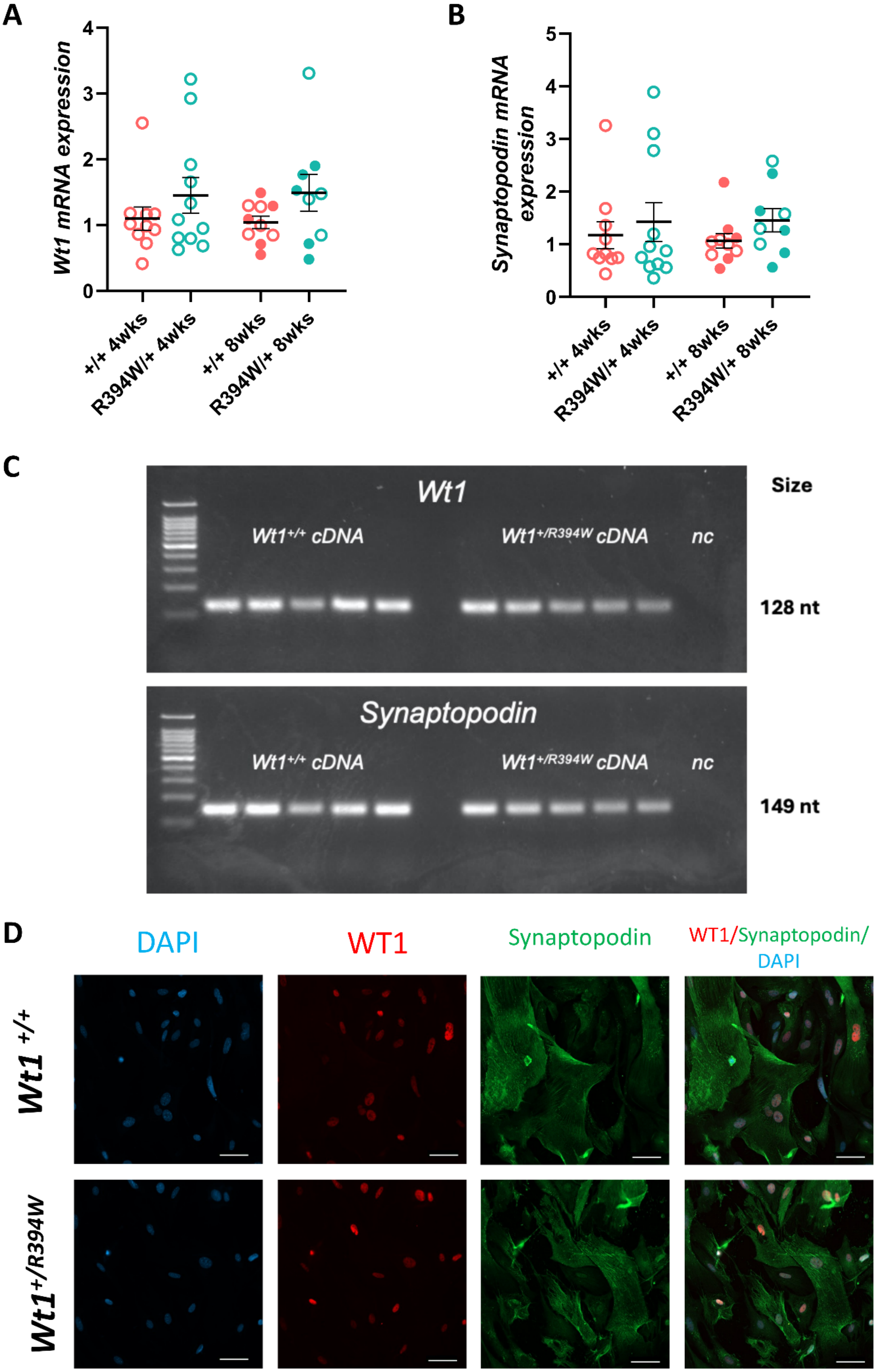
A
Wt1 mRNA expression
0
1
2
3
4
+/+ 4wks
R394W/+ 4wks
+/+ 8wks
R394W/+ 8wks
B
Synaptopodin mRNA expression
0
1
2
3
4
5
+/+ 4wks
R394W/+ 4wks
+/+ 8wks
R394W/+ 8wks
C
Wt1
Wt1+/+ cDNA
Wt1+/R394W cDNA
nc
Size
128 nt
Synaptopodin
Wt1+/+ cDNA
Wt1+/R394W cDNA
nc
149 nt
D
DAPI
WT1
Synaptopodin
WT1/Synaptopodin/DAPI
Wt1 +/+
Wt1+/R394W

Fig. S1 Primary podocyte characterization. (**A**) RT-qPCR data comparing *Wt1* and (**B)** *synaptopodin* mRNA expression in primary podocyte samples isolated from *Wt1*$^{+/+}$ and *Wt1*$^{+/R394W}$ mice at 4 (*Wt1*$^{+/+}$ *n=10* and *Wt1*$^{+/R394W}$ *n=11*) and 8 (*Wt1*$^{+/+}$ *n=10* and *Wt1*$^{+/R394W}$ *n=9*) weeks of age. *Gapdh* used as housekeeping gene. Data represents mean ± SEM. (one-way ANOVA with Tukey's multiple comparisons, no significant difference between genotype at both time points). Filled circles represent five representative samples loaded into (**C**) gels showing PCR products validating *Wt1* (128 nucleotides) and *synaptopdin* (149 nucleotides) expression in *Wt1*$^{+/+}$ and *Wt1*$^{+/R394W}$ primary podocytes. (**D**) Representative images of primary murine podocytes isolated from 8-week-old *Wt1*$^{+/+}$ and *Wt1*$^{+/R394W}$ mice stained for podocyte specific markers WT1 (red) and synaptopodin (green), with DAPI used to label nuclei (blue); composite image shown. Scale bars represent 5 µm.

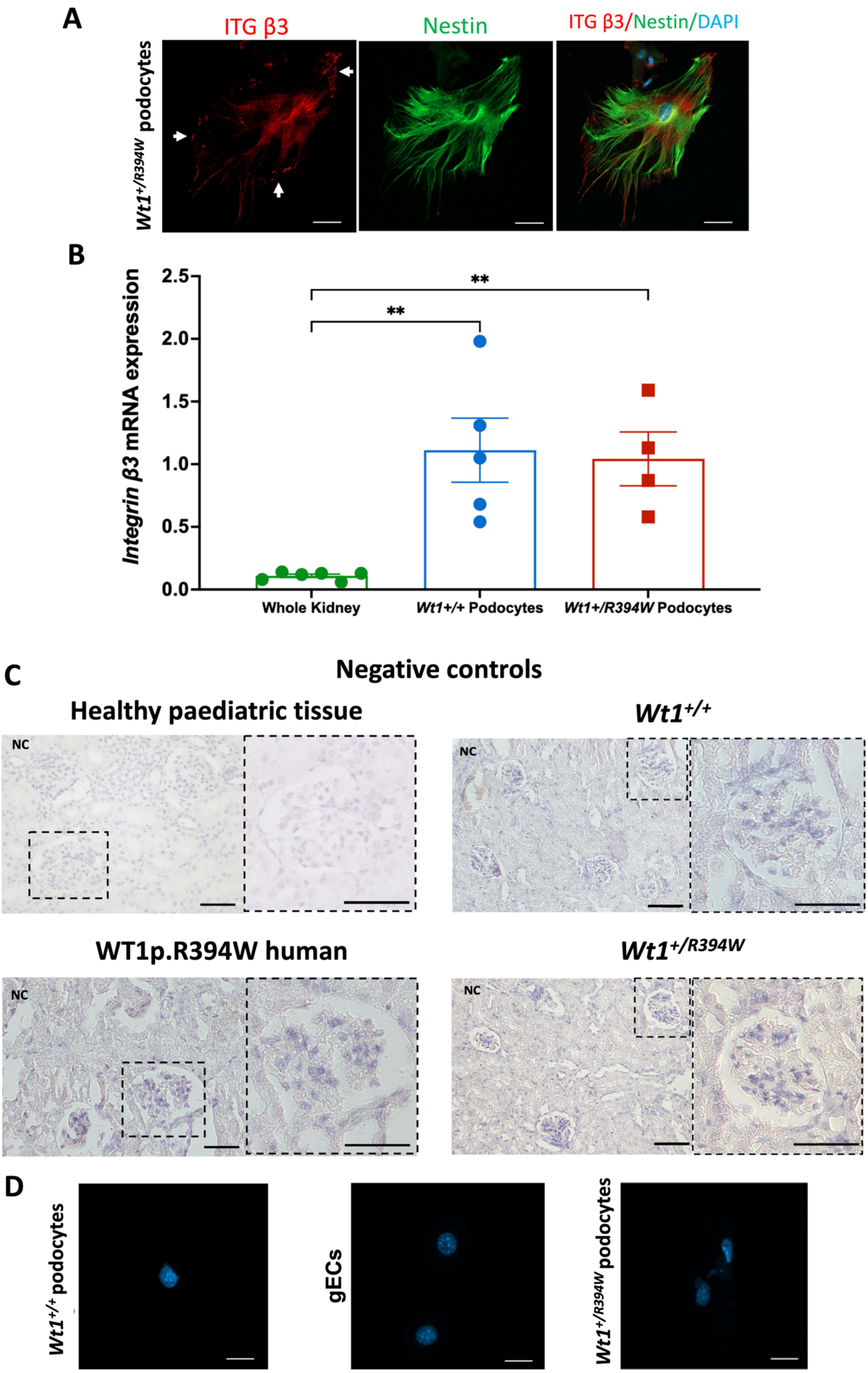
A
ITG β3
Nestin
ITG β3/Nestin/DAPI
Wt1+/R394W podocytes
B
Integrin β3 mRNA expression
2.5
2.0
1.5
1.0
0.5
0.0
**
**
Whole Kidney
Wt1+/+ Podocytes
Wt1+/R394W Podocytes
C
Negative controls
Healthy paediatric tissue
Wt1+/+
NC
WT1p.R394W human
Wt1+/R394W
D
Wt1+/+ podocytes
gECs
Wt1+/R394W podocytes

1172

Fig. S2 Integrin αvβ expression localised and enriched in podocytes compared to the whole kidney in *Wt1*$^{+/R394W}$ *mice.* (**A**) Representative images of primary murine podocytes isolated from *Wt1*$^{+/R394W}$ mice stained for integrin β3 (red) and podocyte skeletal marker nestin (green). DAPI used to label nuclei (blue) and composite image shown. White arrows highlighting plasma membrane localisation of β3; scale bars represent 5 µm. (**B**) qPCR data comparing *β3* mRNA expression in healthy whole kidney ($n = 6$), primary podocyte samples isolated from *Wt1*$^{+/+}$ ($n = 5$) and *Wt1*$^{+/R394W}$ mice ($n = 4$). *Gapdh* used as housekeeping gene. Data represents mean ± SEM (one-way ANOVA with Tukey's multiple comparisons, $^{**}P \leq 0.01$). (**C**) Negative controls (**NC**) omitting primary anti-integrin β3 antibody for healthy paediatric donor, WT1p.R394W human biopsy, *Wt1*$^{+/+}$ *mice*, and *Wt1*$^{+/R394W}$ mice; scale bars represent 50 µm. (**D**) Secondary antibody only treated primary murine podocytes, gECs and *Wt1*$^{+/R394W}$ podocytes used as negative control for integrin β3 staining; scale bars represent 5 µm.

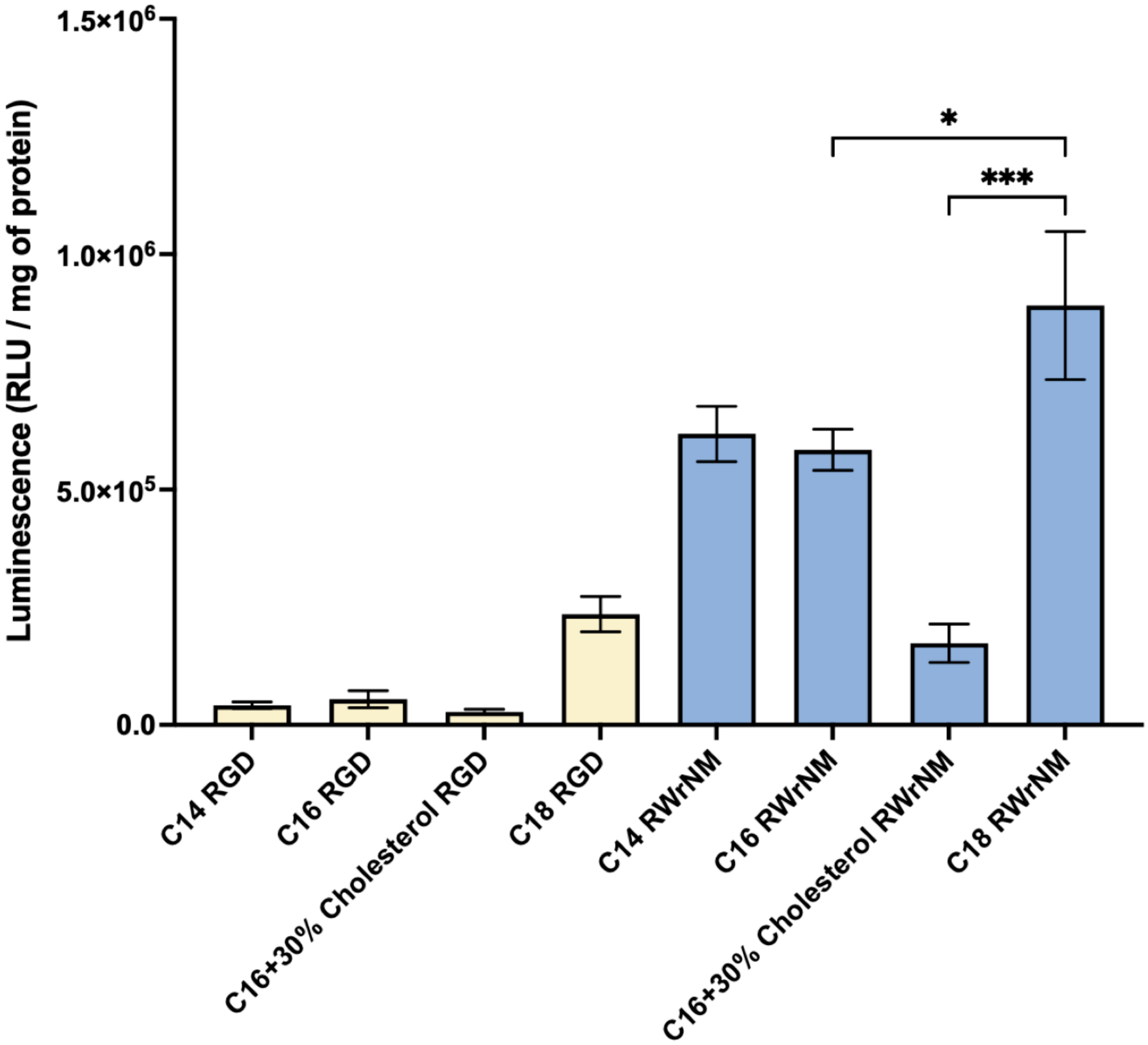

Fig. S3 Effect of cationic liposome formulation on primary murine podocyte transfection. Mean luminescence data (RLU / mg of protein) for primary murine podocytes treated with nanocomplexes composed of different liposome formulations and RGD or RWrNM *(n = 6 per group)*. Data represents mean ± SEM (one-way ANOVA with Tukey's multiple comparisons, *$P \leq 0.05$ and ***$P \leq 0.001$).

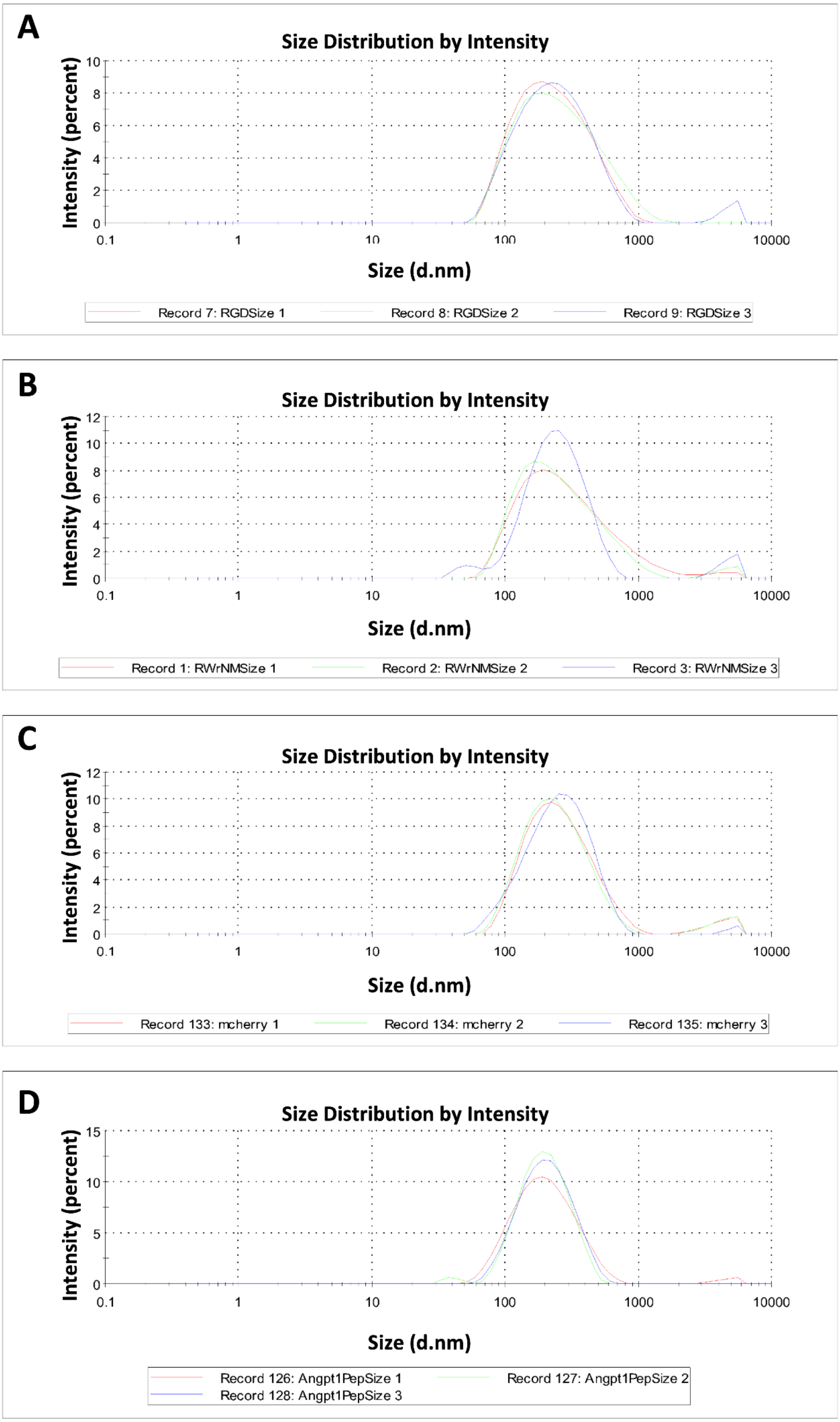
A
Size Distribution by Intensity
Intensity (percent)
Size (d.nm)
Record 7: RGDSize 1
Record 8: RGDSize 2
Record 9: RGDSize 3
B
Size Distribution by Intensity
Intensity (percent)
Size (d.nm)
Record 1: RWrNMSize 1
Record 2: RWrNMSize 2
Record 3: RWrNMSize 3
C
Size Distribution by Intensity
Intensity (percent)
Size (d.nm)
Record 133: mcherry 1
Record 134: mcherry 2
Record 135: mcherry 3
D
Size Distribution by Intensity
Intensity (percent)
Size (d.nm)
Record 126: Angpt1PepSize 1
Record 127: Angpt1PepSize 2
Record 128: Angpt1PepSize 3

Fig. S4 Size distribution of mRNA lipid nanocomplexes. (**A**) Intensity size distribution versus particle diameter in nanometres (d.nm) for Luciferase mRNA cyclic RGD and (**B**) RWrNM nanocomplexes, (**C**) mCherry and (**D**) *Angpt1* mRNA RWrNM nanocomplexes made with fresh sonicated C18 DOTMA:DOPE lipid. Three colours represent repeat measurements of the same sample. Single peaks indicate homogenous nanocomplexes with low polydispersity index (PDI).

A

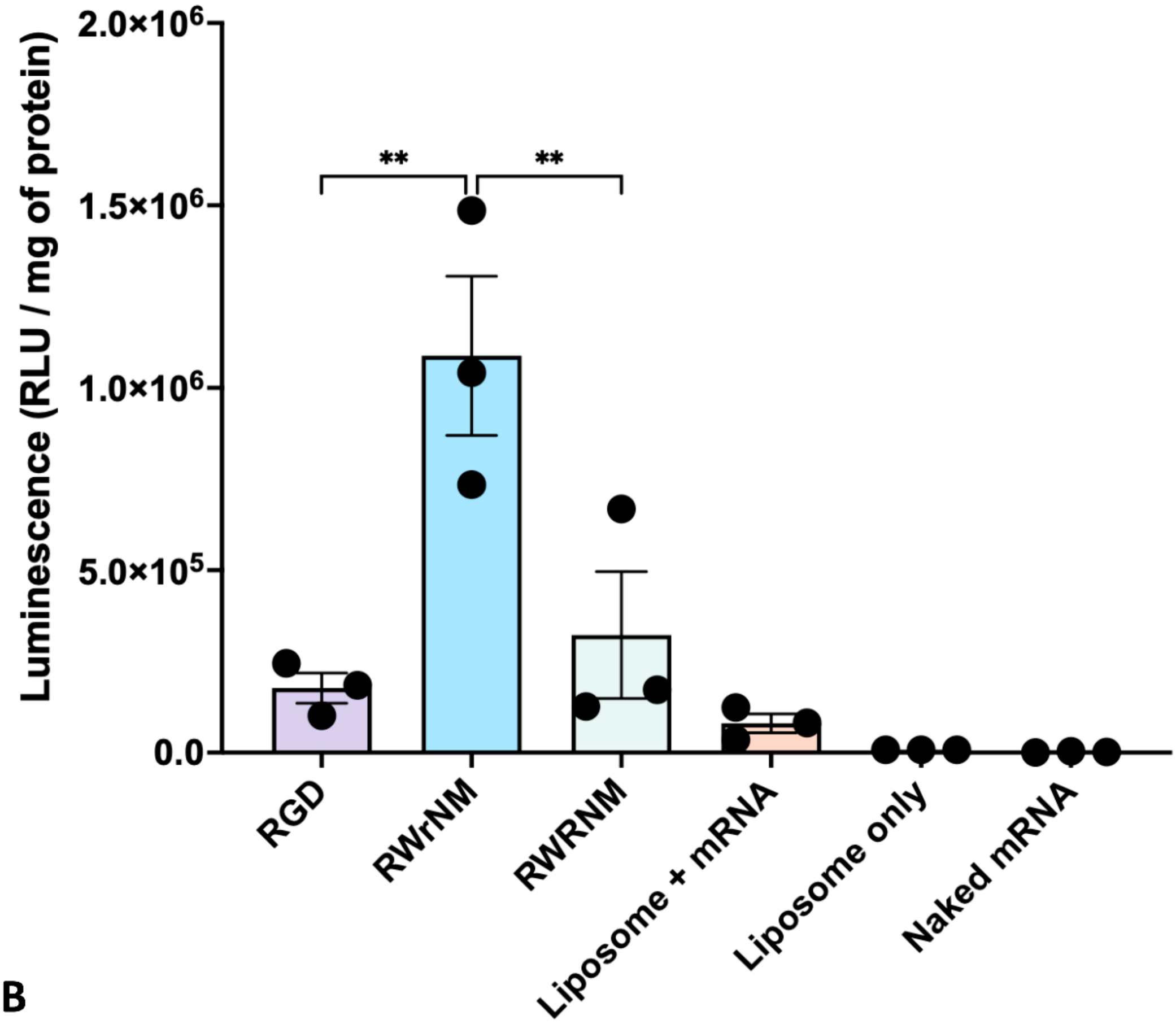
2.0×10⁶
1.5×10⁶
1.0×10⁶
5.0×10⁵
0.0
Luminescence (RLU / mg of protein)
**
**
RGD
RWrNM
RWRNM
Liposome + mRNA
Liposome only
Naked mRNA

B

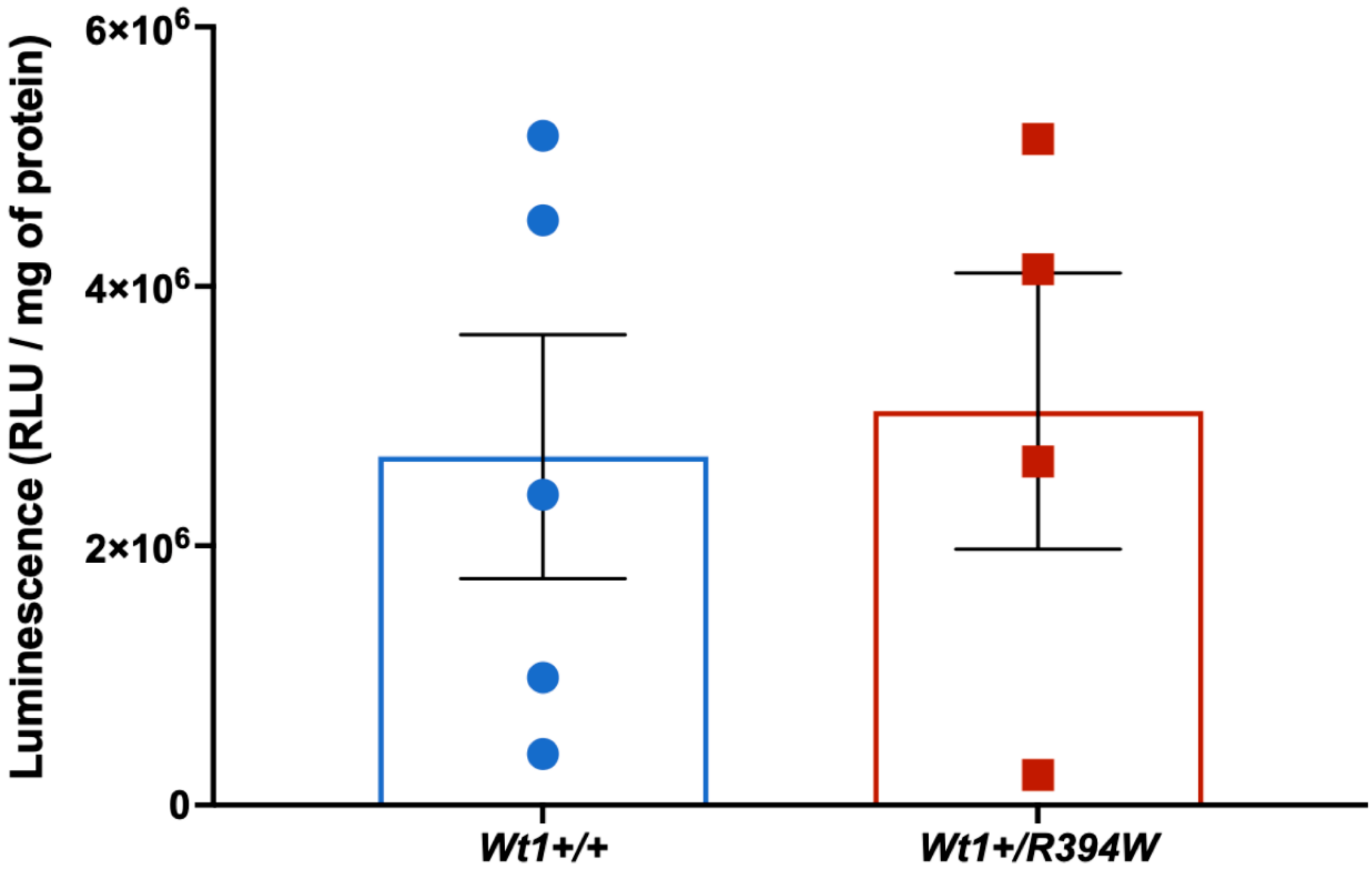
6×10⁶
4×10⁶
2×10⁶
0
Luminescence (RLU / mg of protein)
Wt1+/+
Wt1+/R394W

1196

Fig. S5 RWrNM nanocomplexes elicit highest uptake in *Wt1*$^{+/R394W}$ podocytes with no significant difference compared to wildtype controls. (**A**) Mean luminescence data (RLU / mg of protein) for primary murine *Wt1*$^{+/R394W}$ podocytes treated with luciferase mRNA nanocomplexes to compare cyclic RGD, RWrNM and control RWRNM targeting peptides from three independent experiments. Control groups include no targeting peptide (liposome + mRNA), liposome only and naked mRNA. For each experiment, the podocytes were plated as an *(n = 6)* per group for the luciferase assay, the average of which is plotted here as an individual experiment.. Data represents mean ± SEM (one-way ANOVA with Tukey's multiple comparisons, **P≤0.05, **P≤0.01).* (**B**) Mean luminescence data following optimal RWrNM transfection in *Wt1*$^{+/+}$ *(n=5) and Wt1*$^{+/R394W}$ *(n=4)* primary podocytes. Each data point represents the mean of 1 animal from 3 independent experiments. Data represents mean ± SEM (unpaired t-test).

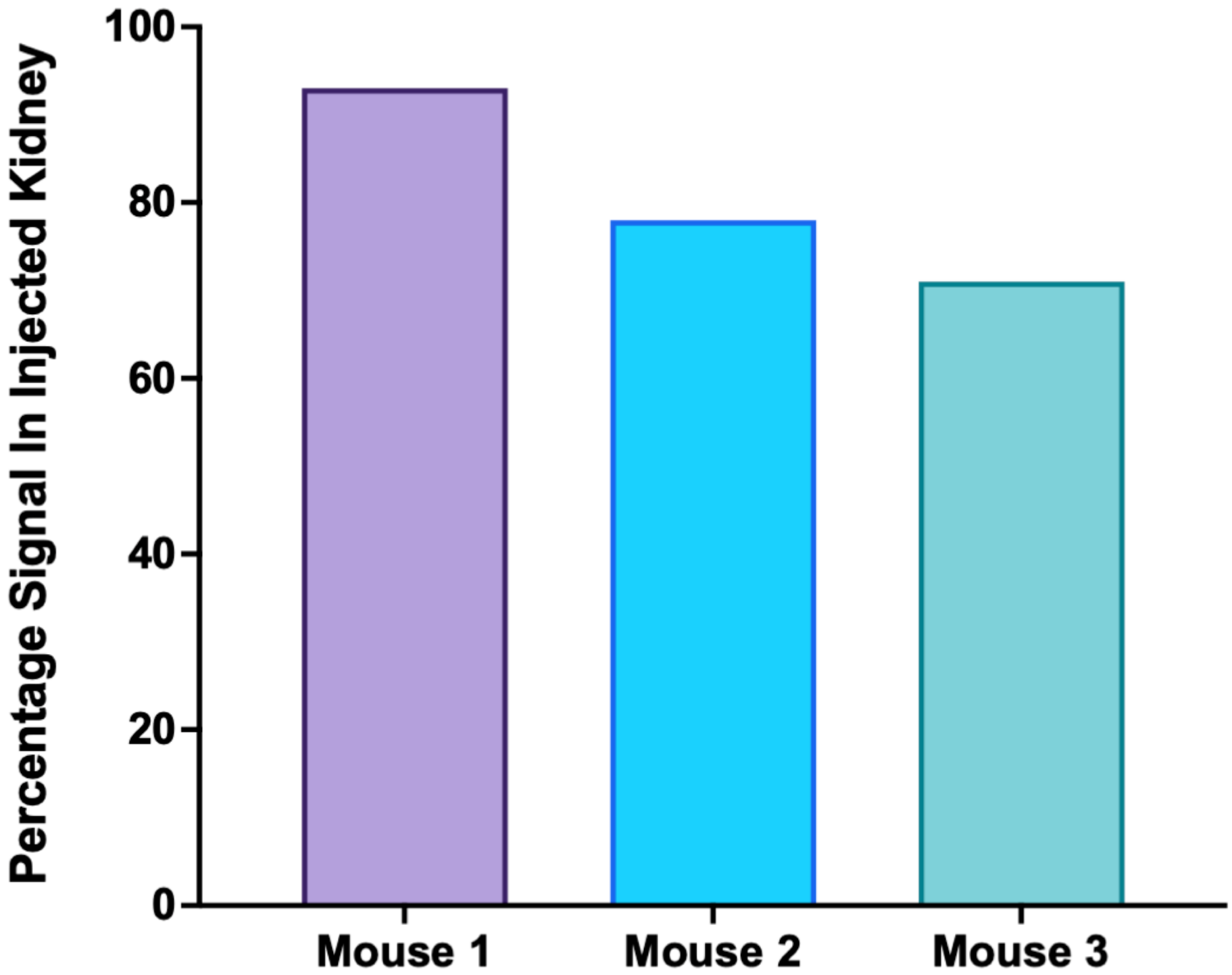

Fig. S6 Ultrasound guided renal artery injection leads to high kidney specificity of RWrNM nanocomplexes. Percentage signal in injected kidney of three 4week-old female CD1 nude mice injected (with 20 µg luciferase mRNA nanocomplexes) under ultrasound guidance directly into the renal artery of the right kidney. Regions of interest were drawn over the right kidney and the whole body to calculate percentage signal in injected kidney, plotted for each mouse.

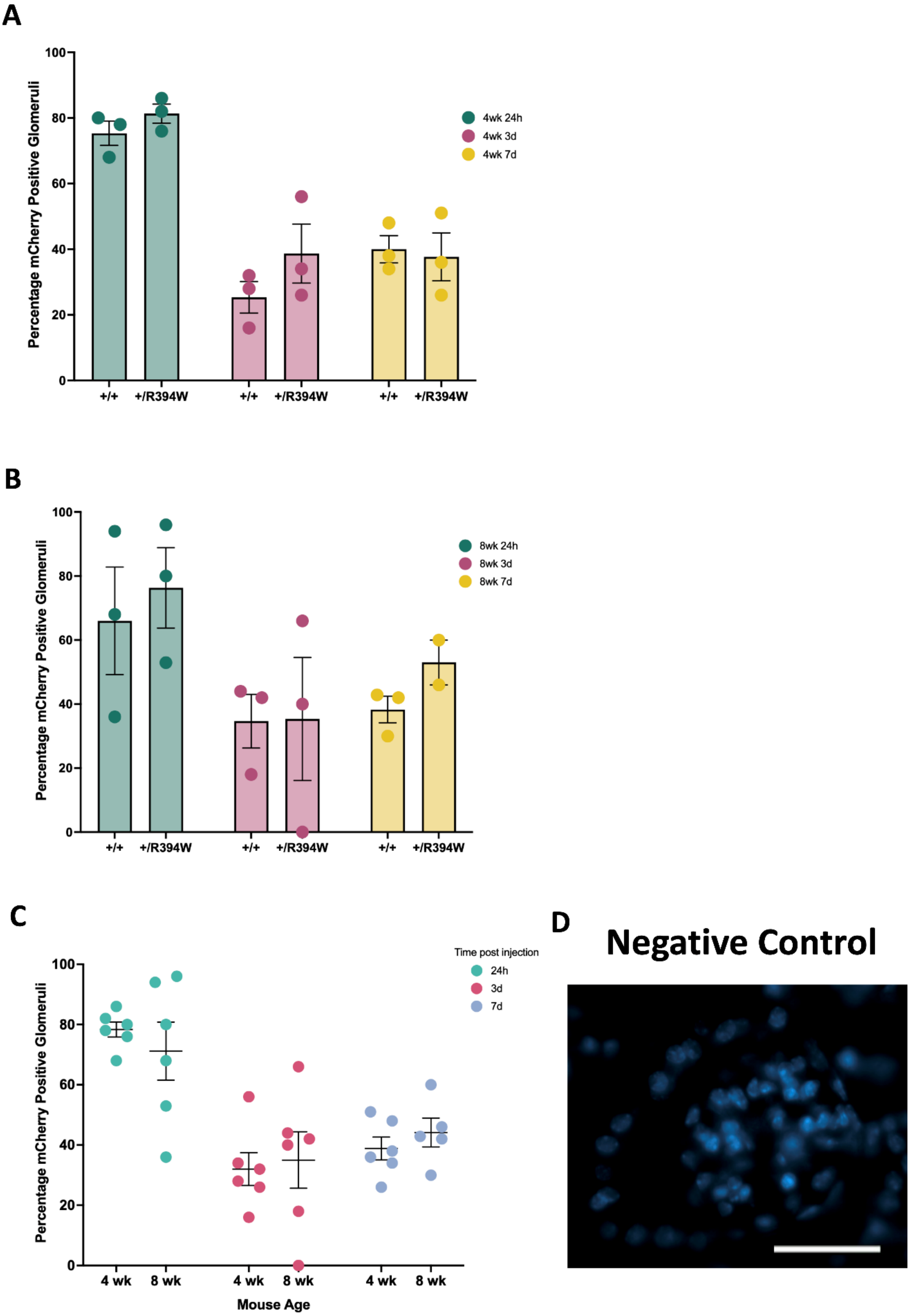
A
Percentage mCherry Positive Glomeruli
100
80
60
40
20
0
+/+
+/R394W
+/+
+/R394W
+/+
+/R394W
4wk 24h
4wk 3d
4wk 7d
B
Percentage mCherry Positive Glomeruli
100
80
60
40
20
0
+/+
+/R394W
+/+
+/R394W
+/+
+/R394W
8wk 24h
8wk 3d
8wk 7d
C
Percentage mCherry Positive Glomeruli
100
80
60
40
20
0
4 wk
8 wk
4 wk
8 wk
4 wk
8 wk
Mouse Age
Time post injection
24h
3d
7d
D
Negative Control

Fig. S7 Glomerular uptake of mCherry mRNA nanocomplexes is not affected by disease state or age. (**A**) Direct comparison in percentage mCherry positive glomeruli in *Wt1*$^{+/+}$ and *Wt1*$^{+/R394W}$ animals at 24 hours, 3 days and 7 days post injection in animals of 4 and (**B**) 8 weeks of age. (**C**) *Wt1*$^{+/+}$ and *Wt1*$^{+/R394W}$ animals were pooled for each time point post injection to assess the effect of age (4 and 8 weeks) on percentage mCherry positive glomeruli. Each data point represents the mean of 50 glomeruli per animal *(n = 3/ group, n* = 2 for 8-week old *Wt1*$^{+/R394W}$ animals at 7 days). Data represents mean ± SEM (two-way ANOVA with Sidak's multiple comparisons). (**D**) Representative secondary antibody only negative control glomerulus for all groups in mCherry immunofluorescence experiment in **Fig. 3**; DAPI in blue; scale bars represent 50 μm.

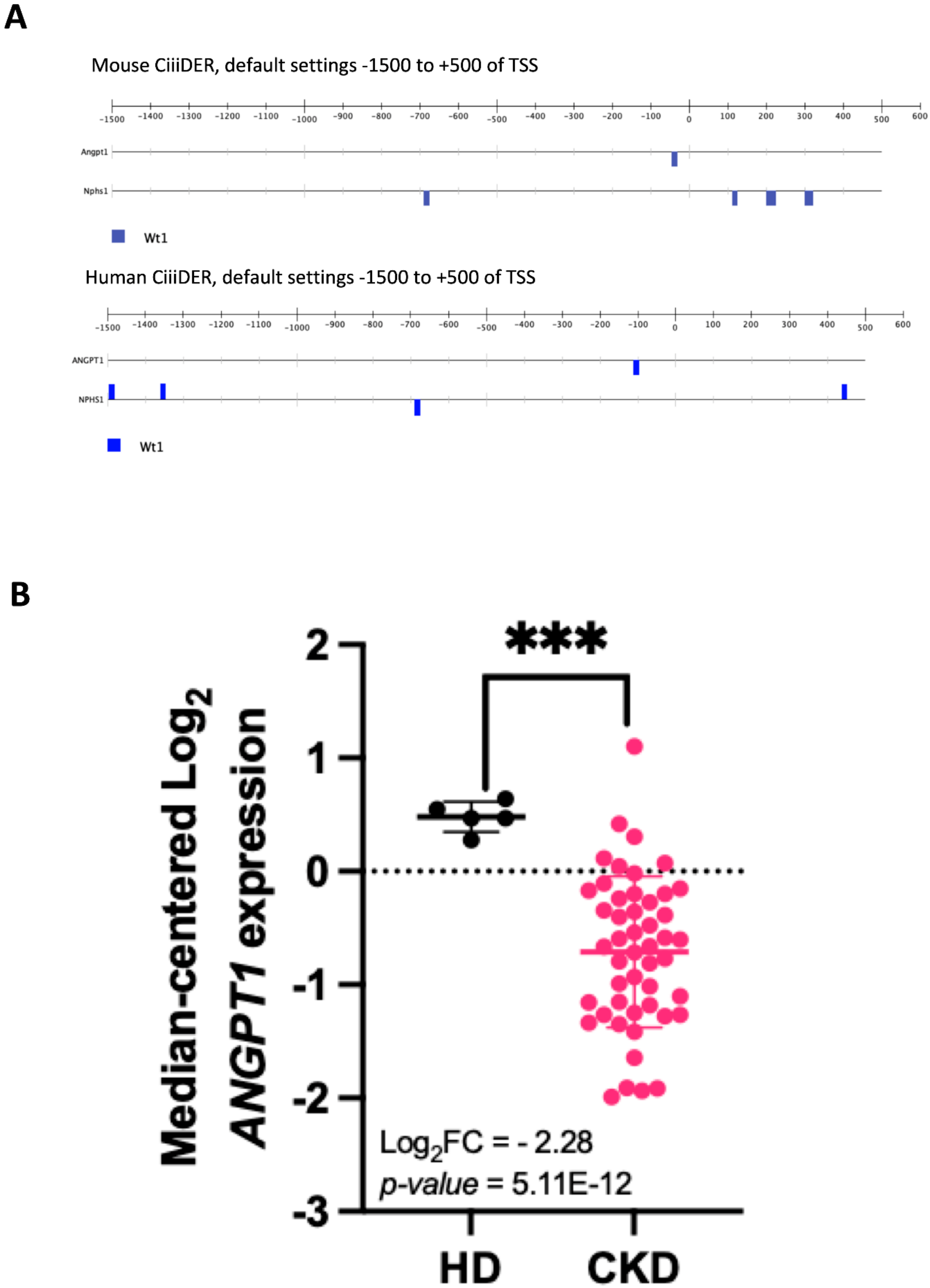

Fig. S8 Predicted WT1 binding motifs in mouse and human *ANGPT1* proximal promoter and significantly reduced *ANGPT1* expression in patients with CKD. (**A**) WT1 motif analysis using CiiiDER within 1.5kb base-pairs downstream and 500 base-pairs upstream of the transcriptional start site using the WT1 motif (MA1627.1)

defined by JASPAR 2022 show predicted WT1 binding motifs in both the mouse and human *ANGPT1* proximal promotors (designated by a blue box). The same region of *NPHS1* (encoding Nephrin), which contains a molecularly validated binding motif for WT1 *(67)*, was included as a positive control of the predicted approach. (**B**) *ANGPT1* is downregulated in human kidney biopsies from patients with CKD (*n* = 48) compared to healthy donors (HD) (*n* = 5) *(71)*. Fold-change and *P*-value indicated on graph and data represents mean ± SEM (unpaired t-test, ***P≤0.001).

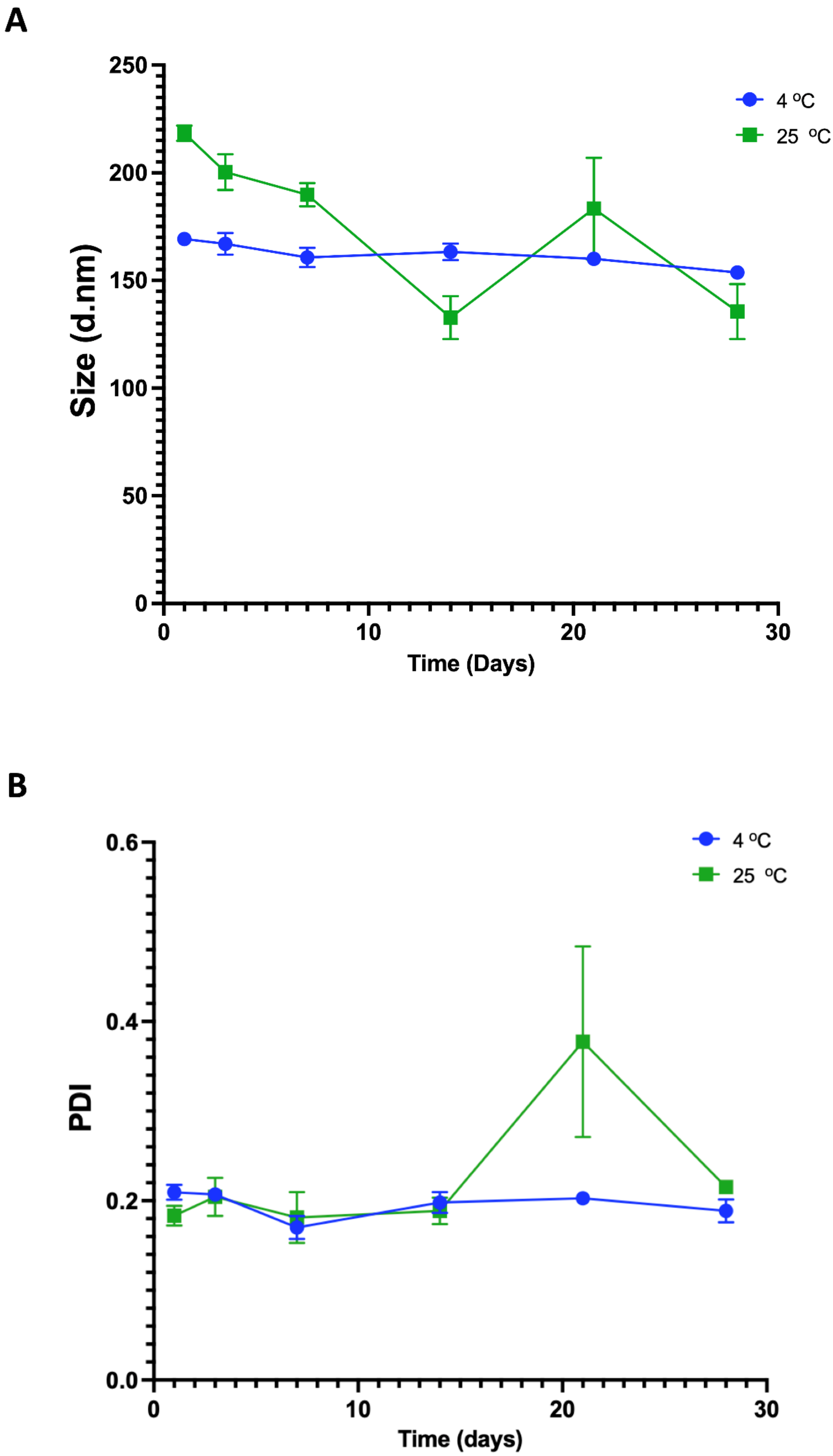
A
Size (d.nm)
250
200
150
100
50
0
0
10
20
30
Time (Days)
4 ºC
25 ºC
B
PDI
0.6
0.4
0.2
0.0
0
10
20
30
Time (days)
4 ºC
25 ºC

1241

Fig. S9 *Angpt1* nanocomplexes are stable for one month at 4 ˚C. (**A**) *Angpt1* nanocomplex size (d.nm) and (**B**) polydispersity index (PDI) for samples stored at 4 ˚C and 25 ˚C at one, three, seven, fourteen, twenty-one and twenty-eight days post incubation. Sixteen measurements of each sample size, the mean of which was recorded for three independent experiments. Data represents mean ± SEM.

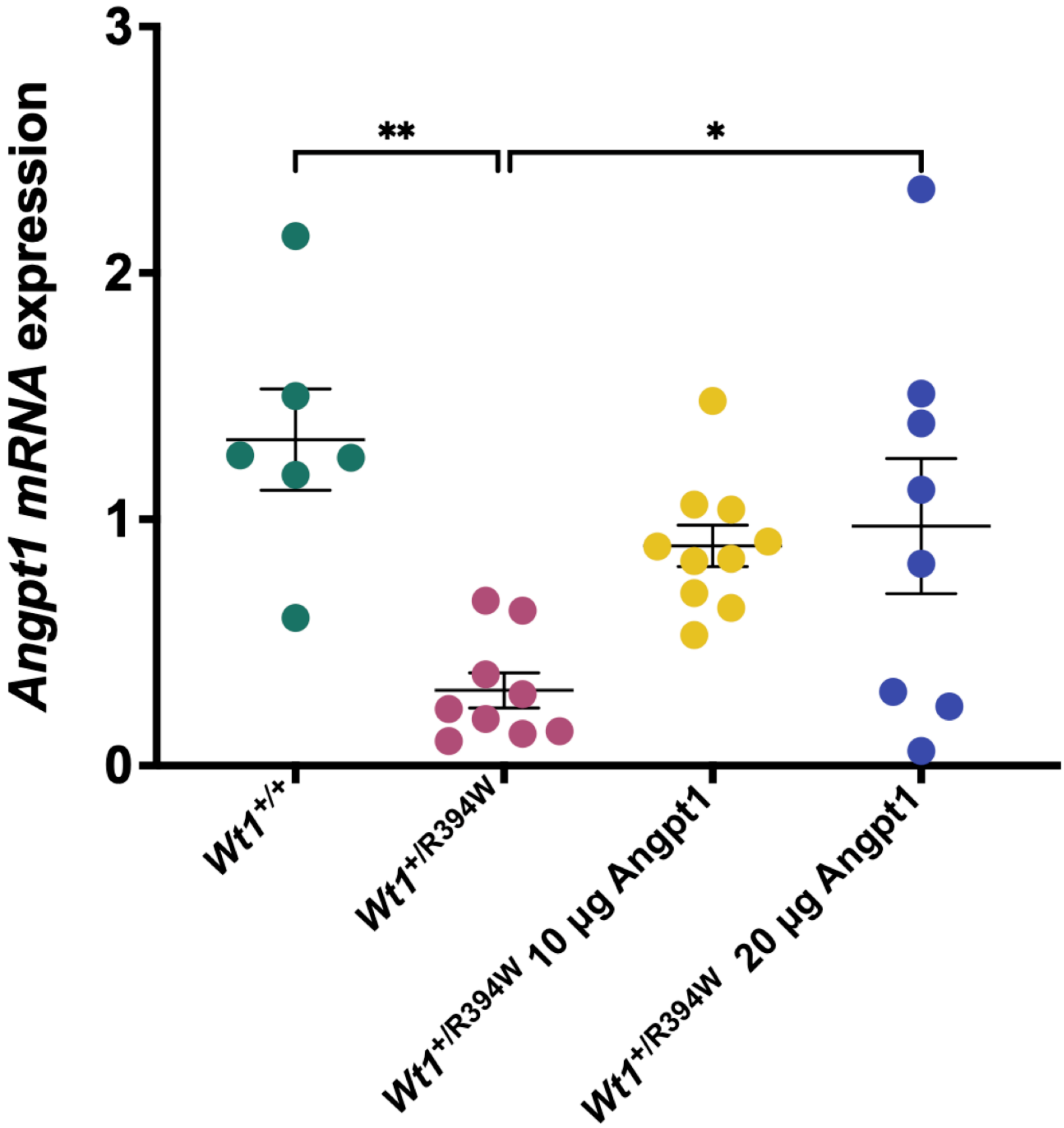

Fig. S10 *Angpt1* mRNA nanocomplexes administered at 20 µg per kidney increases cortical expression of *Angpt1* in *Wt1*$^{+/R394W}$ kidneys. qPCR data for *Angpt1* mRNA expression in the kidney cortex of mice (4 weeks of age) in the following groups: non injected *Wt1*$^{+/+}$ *(n = 3);* non injected *Wt1*$^{+/R394W}$ *(n = 5); Wt1*$^{+/R394W}$ injected with nanocomplexes encapsulating 10 ug of *Angpt1* mRNA per kidney *(n = 5); and Wt1*$^{+/R394W}$ injected with nanocomplexes encapsulating 20 ug of *Angpt1* mRNA per kidney *(n = 4)*. Each data point represents the mean of one kidney. Data represents mean ± SEM (one-way ANOVA with Tukey's multiple comparisons, **P≤0.05 and **P ≤0.01).*

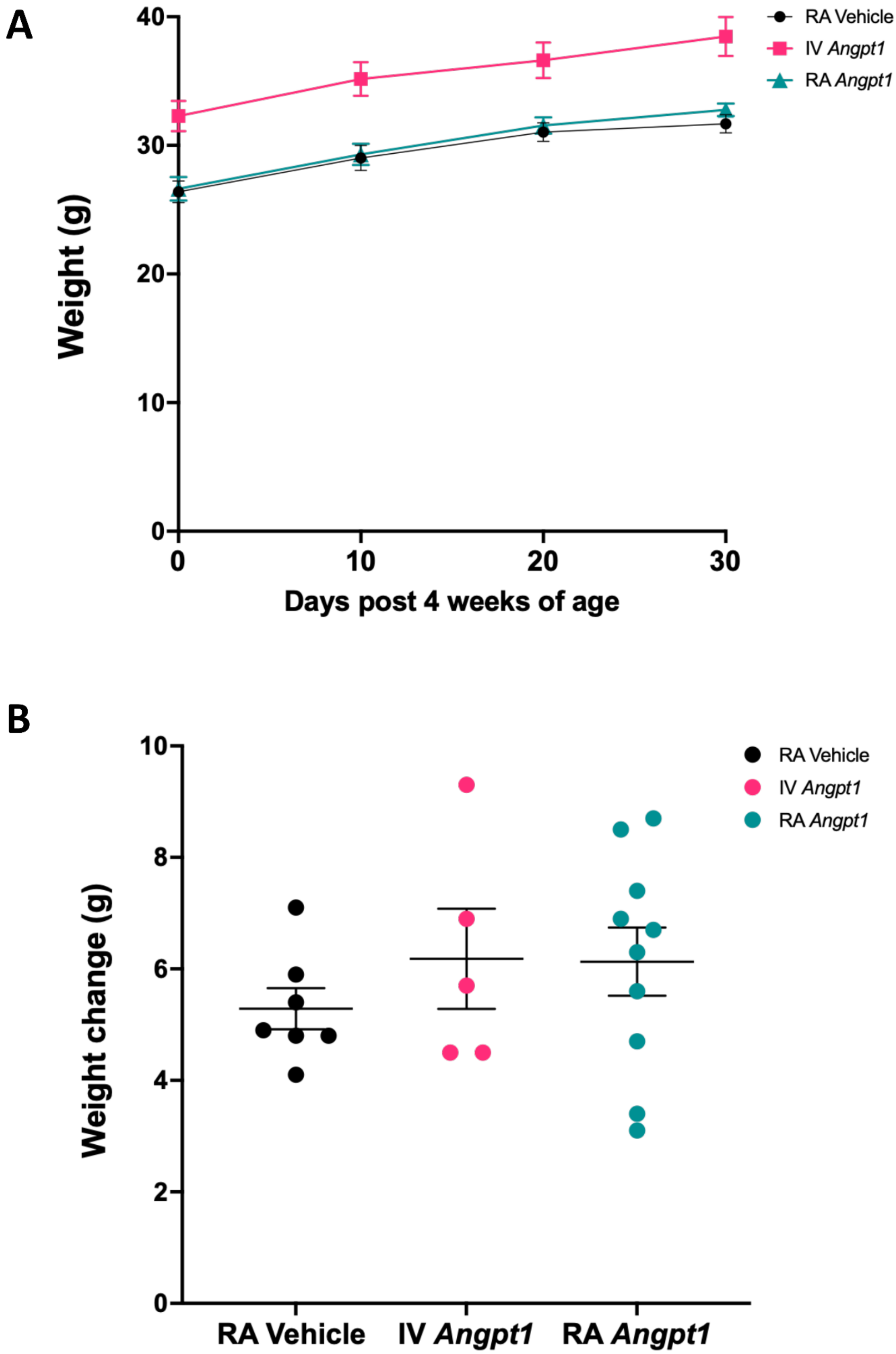
A
Weight (g)
Days post 4 weeks of age
RA Vehicle
IV Angpt1
RA Angpt1
B
Weight change (g)
RA Vehicle
IV Angpt1
RA Angpt1

Fig. S11 Animal weights in *Angpt1* experiment. (**A**) Mean animal weights in grams (g) at 4 weeks of age and every 10 days up to 8 weeks of age in the following treatment groups; vehicle injection via the renal artery (RA Vehicle; $n = 7$), *Angpt1* mRNA administered intravenously (IV *Angpt1*; $n = 5$) and *Angpt1* mRNA delivered via the renal artery (RA *Angpt1*; $n = 10$). Each data point is equivalent to the mean for the group at the specific timepoint. (**B**) Weight change in grams from baseline (day 0) to the termination (day 30) of the experiment for each treatment group. Each data point represents the weight change for one animal. Data represents mean ± SEM (one-way ANOVA with Tukey's multiple comparisons).

A

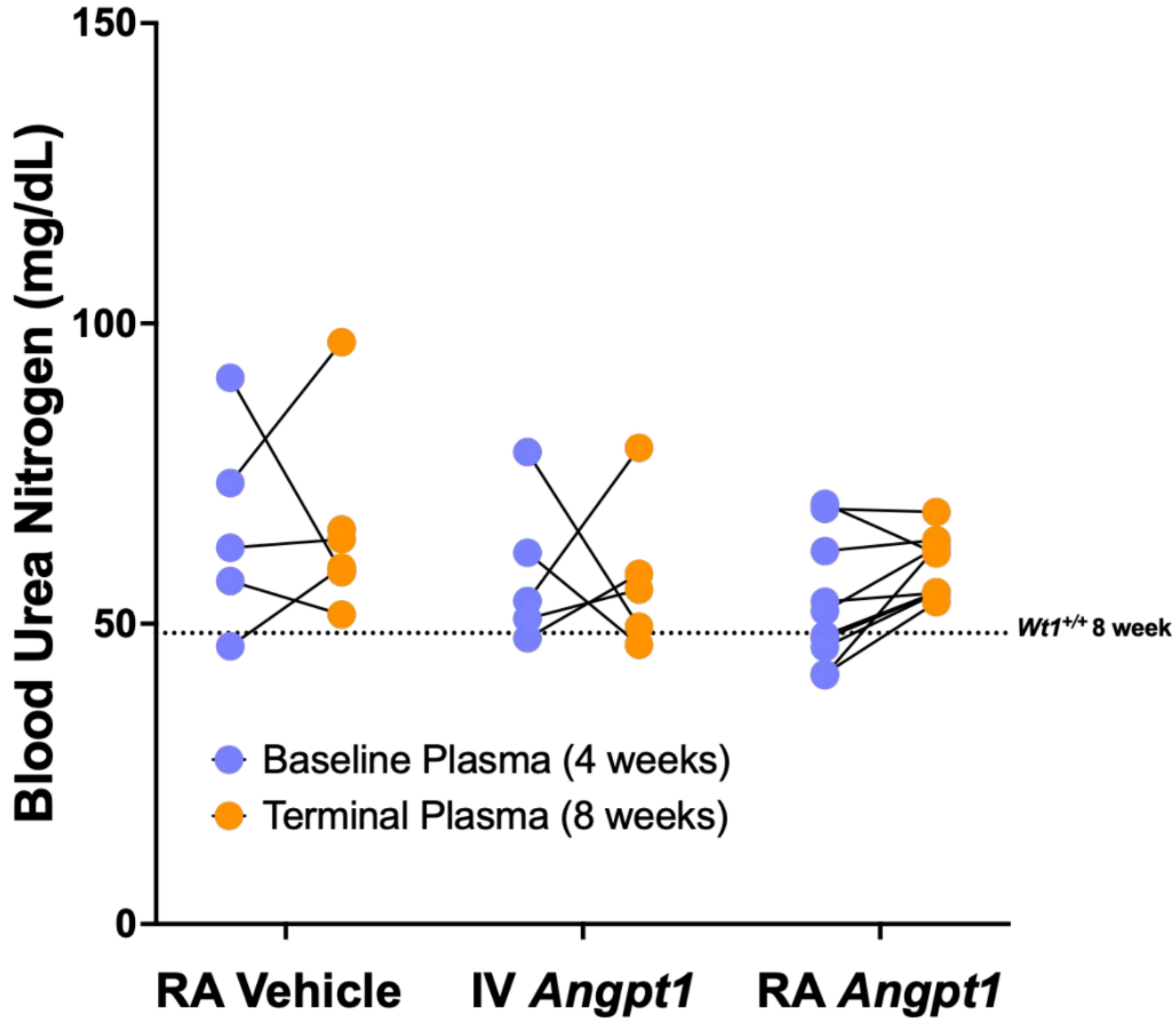
150
100
50
0
Blood Urea Nitrogen (mg/dL)
Wt1+/+ 8 week
Baseline Plasma (4 weeks)
Terminal Plasma (8 weeks)
RA Vehicle
IV Angpt1
RA Angpt1

B

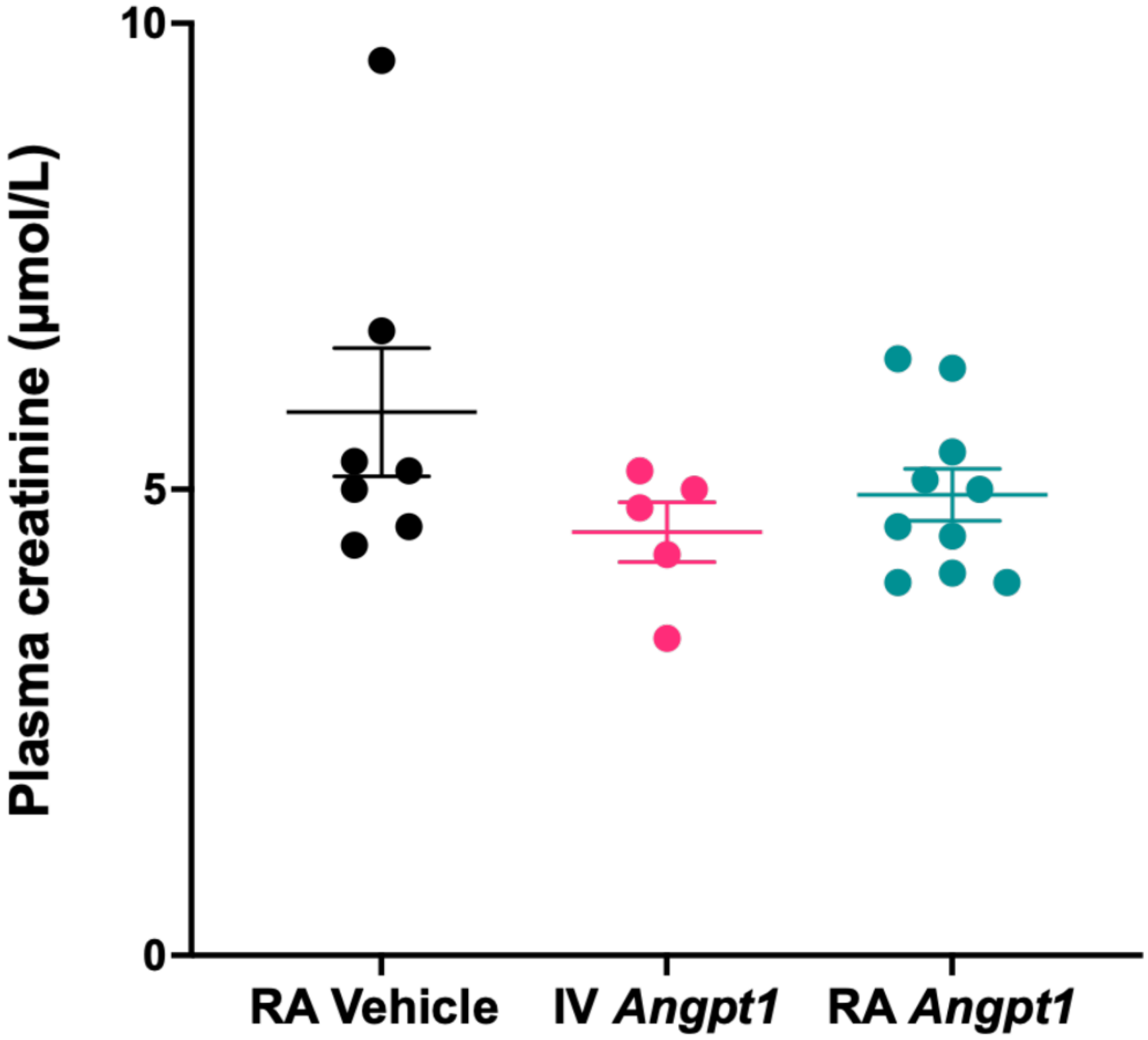
10
5
0
Plasma creatinine (µmol/L)
RA Vehicle
IV Angpt1
RA Angpt1

Fig. S12 ***Angpt1*** **mRNA nanocomplex therapy does not alleviate blood urea nitrogen and plasma creatinine.** (**A**) Blood urea nitrogen (BUN) plasma values at baseline and termination for RA Vehicle (n = 5 baseline and n = *7* termination), IV *Angpt1* (n = 5) and RA *Angpt1* (n = 10) groups. Each data point represents the BUN for an individual animal; paired results linked by lines; mean BUN values at baseline (4 weeks) and termination (8 weeks) were: 66.06 ± 7.61 mg/dL; 58.52 ± 5.59 mg/dL, and 53.10 ± 5.54 mg/dL; 64.97 ± 5.76 mg/dL, and 57.85 ± 3.33 mg/dL; 59.38 ± 1.62 mg/dL for RA vehicle, IV *Angpt1* and RA *Angpt1* respectively (Two-way matched ANOVA, Sidak's multiple comparisons). Dashed line on y-axis indicates mean BUN value for historical $Wt1^{+/+}$ animals at 8 weeks of age (48.45 ± 1.81 mg/dL). (**B**) Plasma creatine measured by liquid chromatography tandem mass spectrometry at termination of the experiment for RA Vehicle (n = 7), IV *Angpt1* (n = *5*) and RA *Angpt1* (n = *10*) groups. Data represents mean ± SEM (one-way ANOVA with Tukey's multiple comparisons).

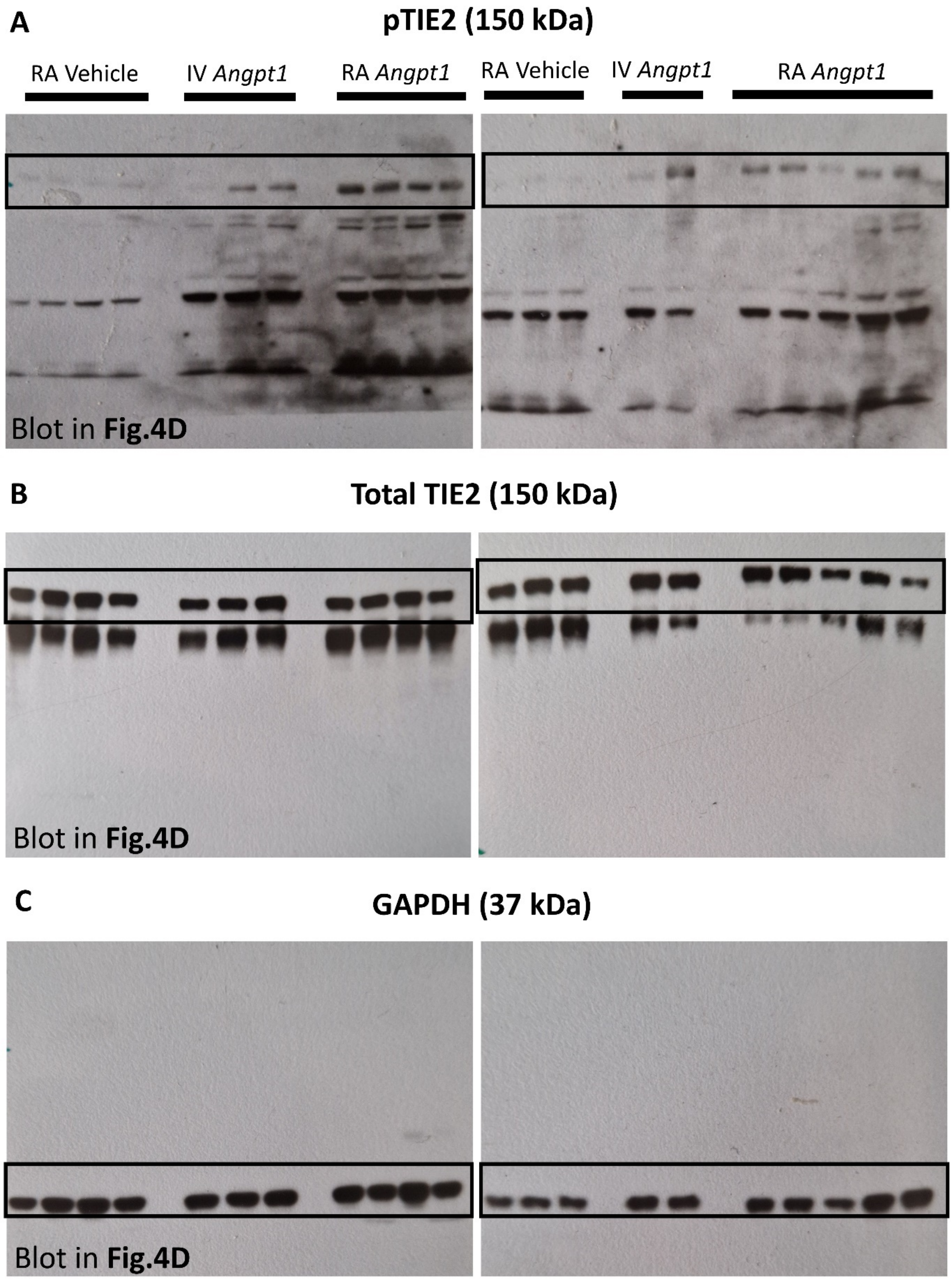
A
pTIE2 (150 kDa)
RA Vehicle
IV Angpt1
RA Angpt1
RA Vehicle
IV Angpt1
RA Angpt1
Blot in Fig.4D
B
Total TIE2 (150 kDa)
Blot in Fig.4D
C
GAPDH (37 kDa)
Blot in Fig.4D

Fig. S13 *Angpt1* mRNA nanocomplexes delivered via the renal artery enhance pTIE2 expression in the kidney cortex *Wt1*$^{+/R394W}$ animals. Full Western blots showing (**A**) pTIE2 (150 kDa), (**B**) total TIE2 (150 kDa) and (**C**) GAPDH (37 kDa) protein expression in the kidney cortex of RA vehicle *(n = 7),* IV *Angpt1 (n = 5)* and RA *Angpt1* (*n = 9,* 1 sample excluded due to low protein concentration) animals. Black boxes highlight bands for each analysed protein and blots used in **Fig. 4C** are indicated.

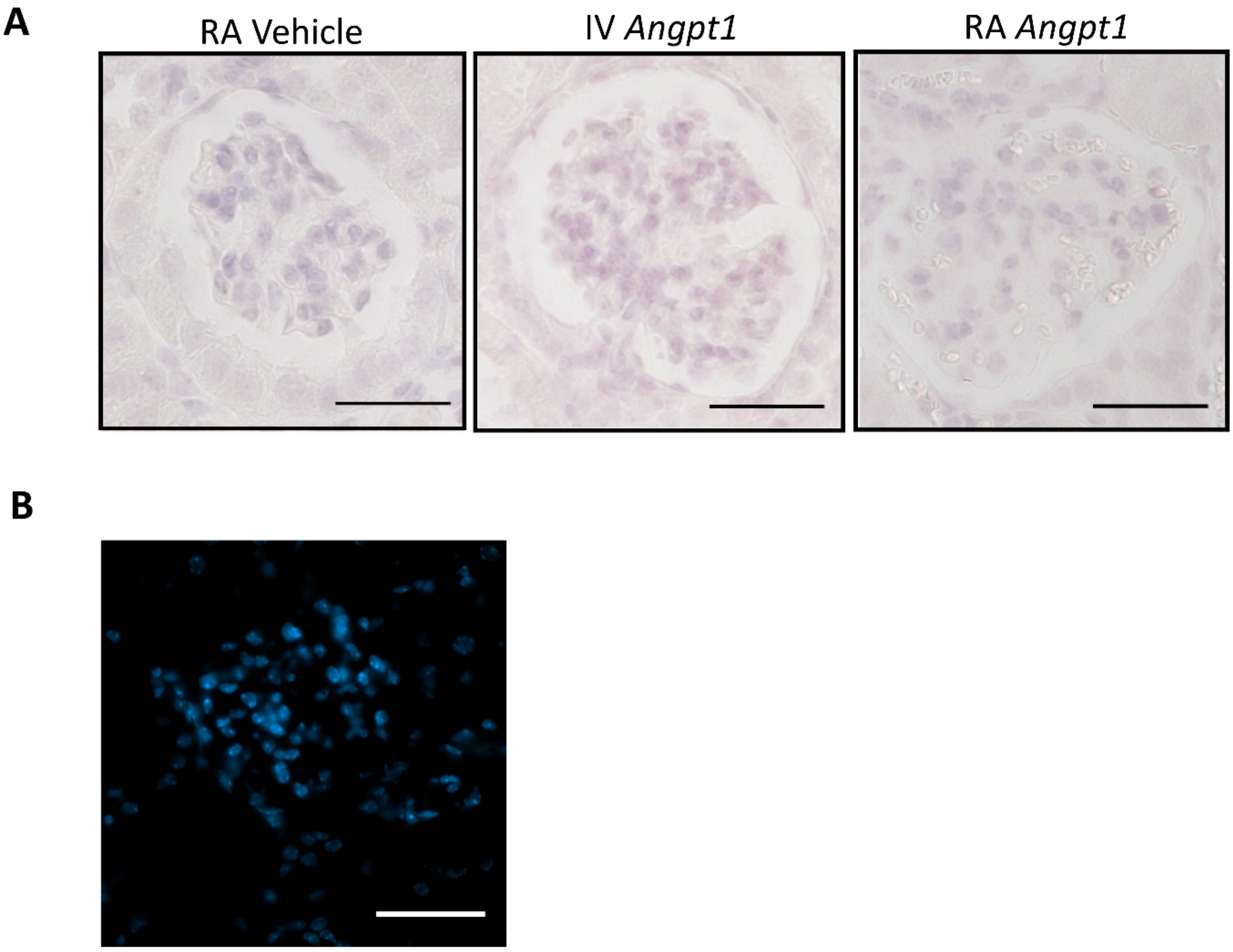

Fig. S14 Negative controls for pTIE2 stained glomeruli and CD31/WT1 stained glomeruli from therapeutic *Angpt1* experiment. (**A**) Negative controls for (**Fig. 4H**), omitting primary pTIE2 antibody for RA vehicle, IV *Angpt1* and RA *Angpt1* groups. (**B**) Representative negative control (secondary only) for CD31/WT1 staining in **Fig. 5C**; scale bars represent 50 µm.

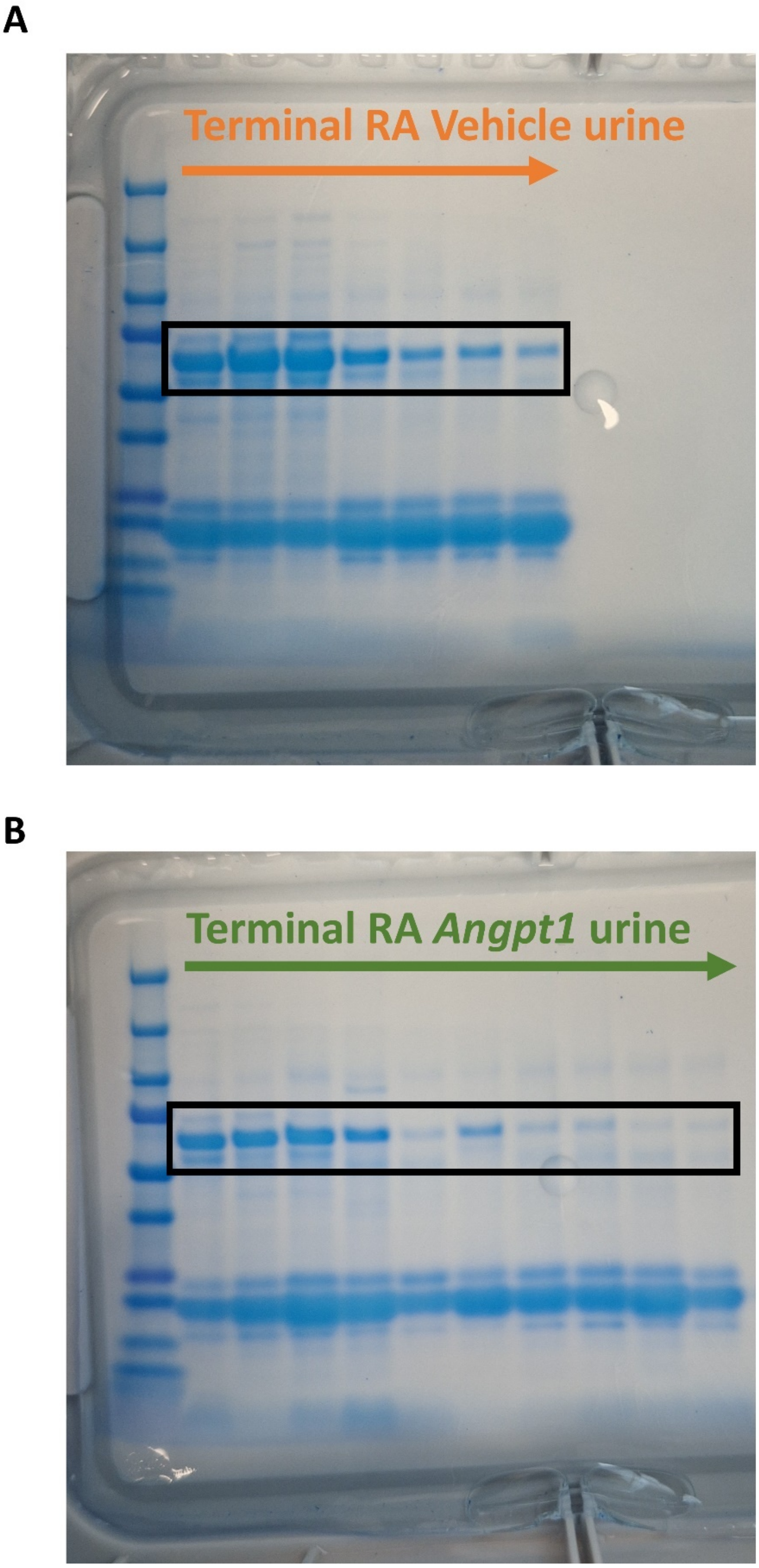

Fig. S15 *Angpt1* nanocomplexes delivered via the renal artery reduces urinary albumin detected by Coomassie blue staining. (**A**) Un-cropped Coomassie blue gels (of **Fig. 5B**) detecting albumin (67 kDa) in terminal RA Vehicle (n = 7) and (**B**) RA *Angpt1* (n = 10) urines.

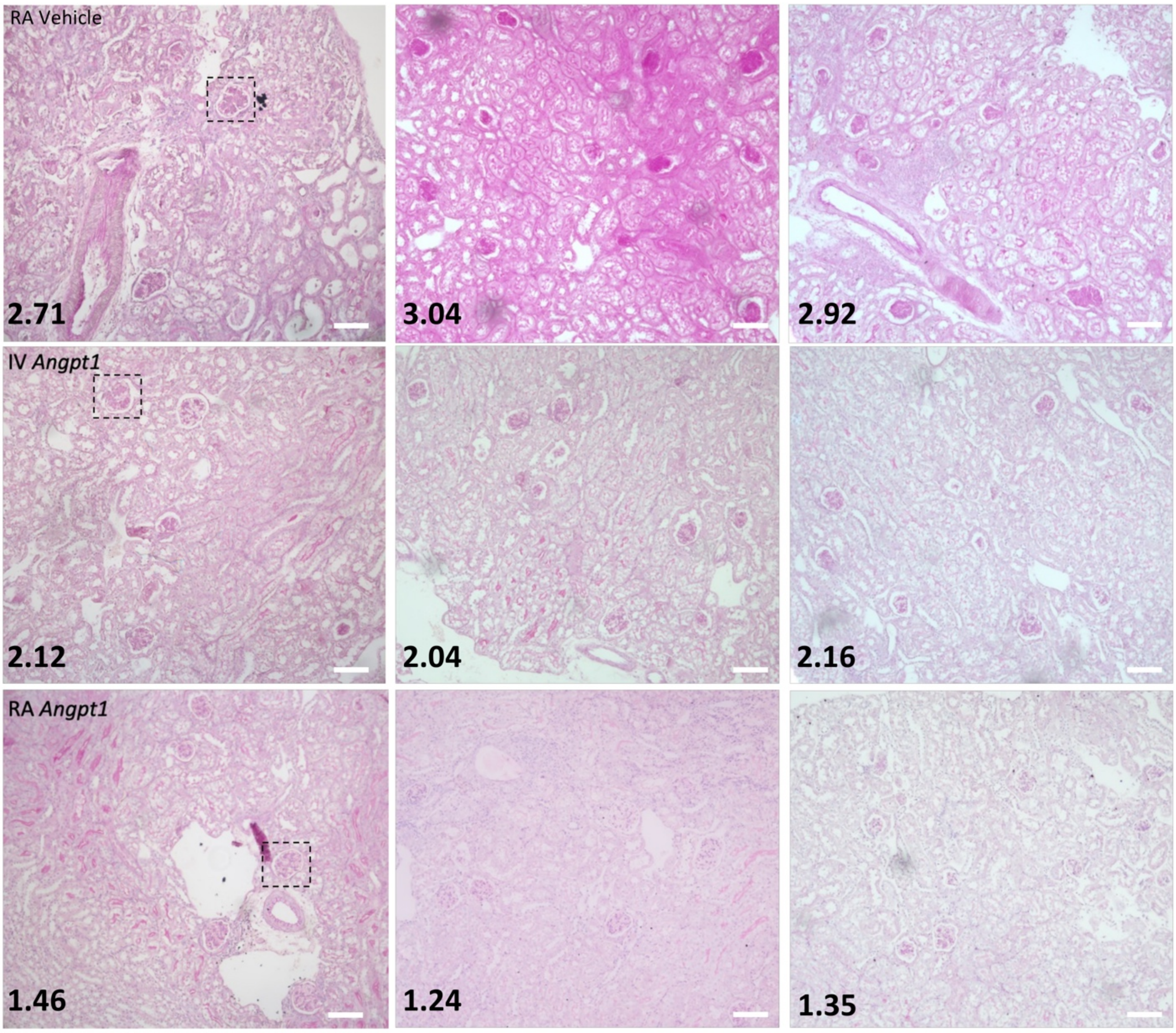

Fig.S16 Low magnification PAS-stained kidney sections. (**A**) Representative PAS-stained kidney sections from three biological repeats and their associated severity index scores for RA vehicle, (**B**) IV *Angpt1*, and (**C**) RA *Angpt1*. Dotted boxes highlight glomeruli shown in **Fig. 5E**. Images captured at 10x magnification. Scale bars represent 50 μm.

Supplementary Tables

Table. S1 Nanocomplex characterisation

Size (d.nm), size peak width (d.nm), polydispersity index (PDI), zeta potential (ZP) (mV), and ZP peak width (mV) for luciferase mRNA (Luc) RGD, Luc RWrNM, mCherry mRNA RWrNM, and *Angpt1* mRNA RWrNM nanocomplexes. Mean of sample measurements (*n* = 3) ± SEM shown

Sample	Size (d.nm)	Size Peak Width (d.nm)	PDI	ZP (mV)	ZP Peak Width (mV)
Luc RGD 1	195.6	168.9	0.296	38.2	4.8
Luc RGD 2	201.1	226.0	0.279	38.4	5.4
Luc RGD 3	209.6	157.3	0.289	37.6	5.2
**Mean ± SD**	**202.1 ± 7.0**	**184.1 ± 36.8**	**0.288 ± 0.009**	**38.1 ± 0.4**	**5.1 ± 0.3**
Luc RWrNM 1	220.1	121.7	0.227	56.6	4.7
Luc RWrNM 2	219.1	218.7	0.264	56.5	3.6
Luc RWrNM 3	221.2	338.4	0.251	55.7	4.7
**Mean ± SD**	**220.1 ± 1.0**	**226.3 ± 108.5**	**0.248 ± 0.019**	**56.3 ± 0.5**	**4.3 ± 0.6**
mCherry RWrNM 1	224.1	167.0	0.274	41.6	4.4
mCherry RWrNM 2	223.9	145.4	0.271	40.4	4.7
mCherry RWrNM 3	234.3	142.9	0.246	42.1	4.7
**Mean ± SD**	**227.4 ± 5.9**	**151.8 ± 13.3**	**0.264 ± 0.015**	**41.4 ± 0.7**	**4.6 ± 0.2**
Angpt1 RWrNM 1	158.1	116.3	0.227	40	4.2
Angpt1 RWrNM 2	147.9	86.7	0.199	40.6	3.9
Angpt1 RWrNM 3	154.7	97.6	0.211	39.6	5.4
**Mean ± SD**	**153.6 ± 5.2**	**100.2 ± 15.0**	**0.212 ± 0.014**	**40.1 ± 0.5**	**4.5 ± 0.8**

Table. S2 Helper and cationic lipids used in this study and their structures

Lipid (alkyl chain length)	Structure
**DOPE (Helper Lipid)**	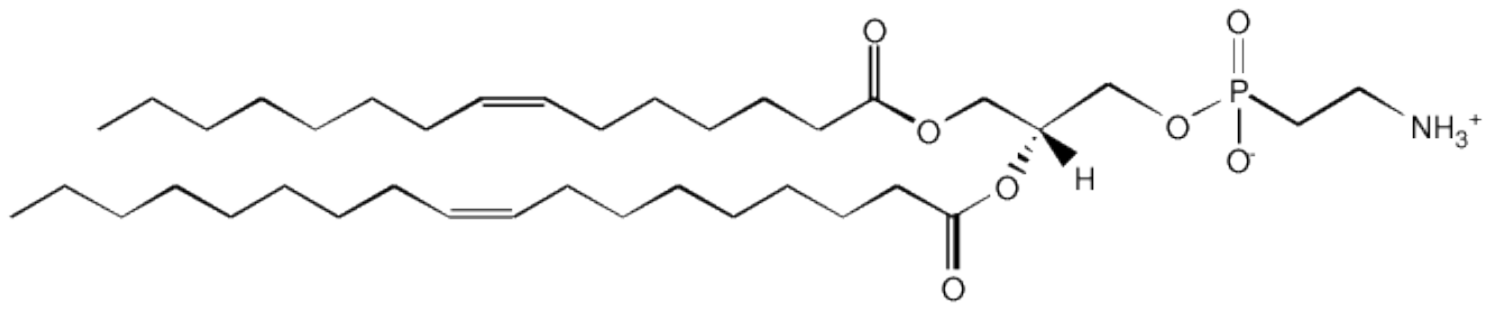
**DTDTMA (C14)**	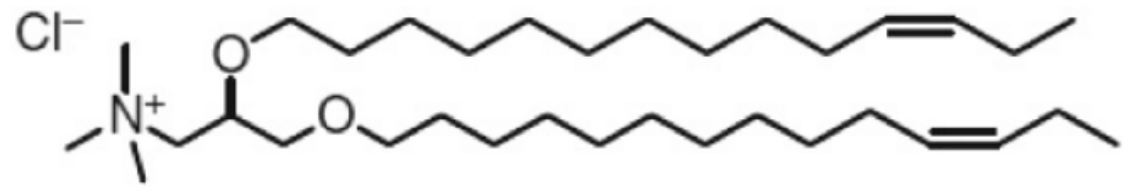
**DHDTMA (C16)**	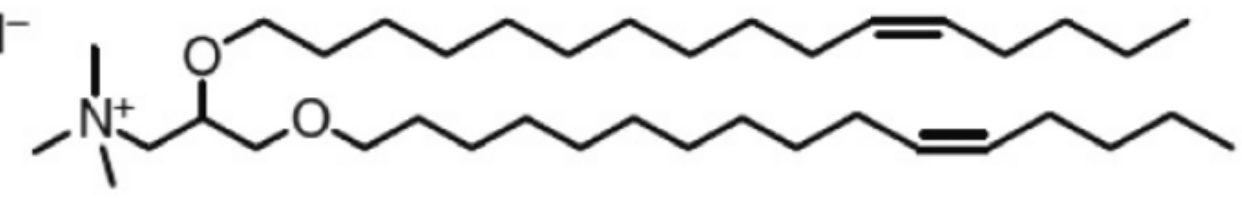
**DOTMA (C18)**	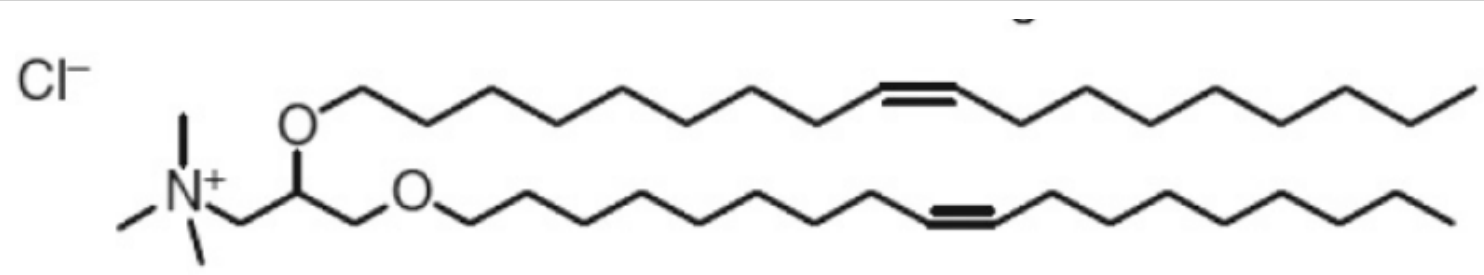
